# Novel Antibacterial Polyglycidols: Relationship between Structure and Properties

**DOI:** 10.3390/polym10010096

**Published:** 2018-01-20

**Authors:** Fabian Marquardt, Cornelia Stöcker, Rita Gartzen, Elisabeth Heine, Helmut Keul, Martin Möller

**Affiliations:** Institute of Technical and Macromolecular Chemistry, RWTH Aachen University and DWI Leibniz-Institute for Interactive Materials, Forckenbeckstr. 50, D-52056 Aachen, Germany; marquardt@dwi.rwth-aachen.de (F.M.); cornelia.stoecker@rwth-aachen.de (C.S.); gartzen@dwi.rwth-aachen.de (R.G.); heine@dwi.rwth-aachen.de (E.H.)

**Keywords:** antimicrobial polymers, functional polyethers, structure-property relationship, multifunctional polyglycidol, post-polymerization functionalization

## Abstract

Antimicrobial polymers are an attractive alternative to low molecular weight biocides, because they are non-volatile, chemically stable, and can be used as non-releasing additives. Polymers with pendant quaternary ammonium groups and hydrophobic chains exhibit antimicrobial properties due to the electrostatic interaction between polymer and cell wall, and the membrane disruptive capabilities of the hydrophobic moiety. Herein, the synthesis of cationic–hydrophobic polyglycidols with varying structures by post-polymerization modification is presented. The antimicrobial properties of the prepared polyglycidols against *E. coli* and *S. aureus* are examined. Polyglycidol with statistically distributed cationic and hydrophobic groups (cationic–hydrophobic balance of 1:1) is compared to (i) polyglycidol with a hydrophilic modification at the cationic functionality; (ii) polyglycidol with both—cationic and hydrophobic groups—at every repeating unit; and (iii) polyglycidol with a cationic–hydrophobic balance of 1:2. A relationship between structure and properties is presented.

## 1. Introduction

Contamination by microorganisms such as bacteria, fungi, and algae is a key issue in medicine [1], pharmaceutical production [2], water purification systems [3], food packaging [4], and various other fields [5,6]. Artificial materials lack defense against microbial growth, allowing microbes to attach to the surface and form a biofilm [7]. One way to prevent the biofilm formation is the usage of disinfectants to keep the surface sterile. Common disinfectants include low molecular weight substances such as alcohols, aldehydes, quaternary ammonium compounds, silver compounds, peroxygens, and bisphenols [8]. However, these classes of antimicrobial substances need to be applied regularly, leading to the development of resistance in the microbial strains [9]. Another way to prevent microbial growth is the coating of surfaces with antimicrobial substances that either kill microorganisms on contact or repel the attachment of microbes [10]. Nevertheless, the leaching of biocides from those coatings causes the same previously mentioned problems. An attractive alternative to low molecular weight biocides are antimicrobial polymers, because they are non-volatile, chemically stable, and can be used as non-releasing additives [11]. These polymers are prepared either by (co-)polymerization of functionalized monomers or by post-polymerization functionalization [12]. Though, the antimicrobial properties are derived from a variety of functionalities such as biguanides [13,14], benzoate esters and benzaldehydes [15], or poly(acrylic acid) [16], the most common active moieties are based on a combination of quaternary ammonium, pyridinium, or phosphonium groups and hydrophobic functionalities [17,18,19]. The proposed and commonly accepted mechanism for these types of polymers features the electrostatic interaction between the cationic moieties and the negatively charged bacterial cell membrane and the disruption of the cell membrane by the hydrophobic groups, leading to leakage and, subsequently, cell death [20,21]. However, due to the heterogeneity and bacterial strain specificity of the cell wall composition, the efficacy of antimicrobial polymers is species-dependent. The outer part of the cell wall of Gram-positive bacteria is composed of about 90% peptidoglycan. In Gram-negative bacteria, the peptidoglycan layer accounts for approximately 20% of the cell envelope, being located between the outer membrane and inner cell membrane. Before reaching the inner cell membrane, polymers interact with the negatively charged components of the outer part of the bacterial cell envelope, e.g., teichoic acids in the thick peptidoglycan layers of Gram-positive bacteria, and phospholipids and lipopolysaccharides in the outer membrane of Gram-negative bacteria [22,23]. The effectiveness of the antimicrobial cationic–hydrophobic polymers is dependent on various factors [24]. One factor is the molecular weight of the polymer. Ikeda et al. synthesized polymethacrylates with pendant biguanide units and various molecular weights and tested the antimicrobial activity against *Staphylococcus aureus*, reaching an optimal activity at molecular weights between 50 and 100 kDa [25]. Kanazawa et al. showed an increase in biocidal activity of poly[tributyl(4-vinylbenzyl)phosphonium chloride] against *S. aureus* with increasing molecular weight [26]. Based on the described mechanism, they assume that a higher molecular weight and a consequential higher charge density enhance the adsorption of the polymers to the cell membrane. A stronger adsorption leads to a stronger disruption of the cell membrane and, thus, to a higher activity of the polymer. However, Lienkamp et al. found that when the molecular weight reaches a threshold value, the efficacy of the polymers decreases [27]. Additionally, Locock et al. reported the reverse effect on guanylated polymethacrylates [28]. The presented polymers showed higher antimicrobial activity at lower molecular weights, proving that the efficacy of antimicrobial polymers does not exclusively depend on their molecular weight. A second factor for the antimicrobial behavior is the alkyl chain length of the hydrophobic moiety. However, the optimal alkyl chain length is different for different types of polymers. Pasquier et al. prepared branched poly(ethylene imine)s with pendant ammonium and alkyl functionalities, ranging from C_6_ to C_16_. An increase in the alkyl chain length lead to an increase in the antimicrobial activity against *E. coli* [29]. The activity was further enhanced by He et al., attaching the hydrophobic chain directly to the ammonium group [30]. The opposite influence of aliphatic side chains was reported by Xu et al. on comb-like ionenes [31]. Here, a decrease in the alkyl chain length lead to an increase in the antimicrobial activity against *E. coli*. On the other hand, Chen et al. prepared poly(propylene imine) dendrimers with alkyl chains ranging from C_8_ to C_16_, showing a parabolic dependence between the biocidal effect and the alkyl chain length, with the highest activity at C_10_ [32].

In the current work, we present the preparation of various antimicrobial polymers based on polyglycidol with quaternary trimethylammonium groups as the cationic moiety and dodecyl chains as the hydrophobic part. Polyglycidol is a highly functional polymer with a hydroxy group in every repeating unit, allowing various further modifications [33,34]. It is non-toxic, soluble in aqueous media, and licensed by the Food and Drug Administration (FDA) [35,36]. The cationic and hydrophobic side chain functionalities were distributed statistically along the polymer backbone (cationic–hydrophobic ratio of 1:1). This functionalized polyglycidol was compared to (i) a polyglycidol modified with hydrophilic hydroxyethyl functionalities at the cationic center; (ii) a polyglycidol with cationic and hydrophobic moieties at every repeating unit; and (iii) a polyglycidol with a cationic–hydrophobic balance of 1:2. All polymers were tested in regard to their antimicrobial properties against *Escherichia coli* and *Staphylococcus aureus* to examine a possible relationship between the structure and the biocidal effect of the polymer.

## 2. Materials and Methods 

### 2.1. Materials

Potassium *tert*-butoxide (1 M solution in THF, Sigma Aldrich Chemie GmbH, Steinheim, Germany), diglyme (≥99%, extra dry, over molecular sieves, Thermo Fisher Acros Organics, Geel, Belgium), pyridine (99.5%, extra dry, over molecular sieves, Thermo Fisher Acros Organics, Geel, Belgium), phenyl chloroformate (>97%, Sigma Aldrich Chemie GmbH, Steinheim, Germany), 4-nitrophenyl chloroformate (>98%, TCI Deutschland GmbH, Eschborn, Germany), 3-(dimethylamino)-1-propylamine (99%, Thermo Fisher Acros Organics, Geel, Belgium), *N*-(3-aminopropyl)diethanolamine (>90%, TCI Deutschland GmbH, Eschborn, Germany), dodecylamine (98%, Sigma Aldrich Chemie GmbH, Steinheim, Germany), 4-dimethylaminopyridine (>98%, Sigma Aldrich Chemie GmbH, Steinheim, Germany), dl-homocysteine thiolactone hydrochloride (98%, abcr GmbH, Karlsruhe, Germany), triethylamine (≥99.5%, anhydrous, Sigma Aldrich Chemie GmbH, Steinheim, Germany), dodecyl acrylate (>98%, TCI Deutschland GmbH, Eschborn, Germany), methyl iodide (>99%, Sigma Aldrich Chemie GmbH, Steinheim, Germany), tetrahydrofuran (99.8%, stabilizer free, extra dry, Thermo Fisher Acros Organics, Geel, Belgium), chloroform (99.9%, extra dry, over molecular sieves, Thermo Fisher Acros Organics, Geel, Belgium), *N*,*N*-dimethylformamide (99.8%, extra dry, over molecular sieves, Thermo Fisher Acros Organics, Geel, Belgium), methanol (≥99.8%, p.a., Th. Geyer CHEMSOLUTE^®^, Renningen, Germany), and dichloromethane (≥99.8%, anhydrous, Sigma Aldrich Chemie GmbH, Steinheim, Germany) were used as received.

The 3-phenyl-1-propanol (99%, Sigma Aldrich Chemie GmbH, Steinheim, Germany) was stirred with calcium hydride for 24 h and then distilled. Ethoxyethyl glycidyl ether (EEGE) was synthesized from 2,3-epoxypropan-1-ol (glycidol, 96%, Sigma Aldrich Chemie GmbH, Steinheim, Germany) and ethyl vinyl ether (99%, Sigma Aldrich Chemie GmbH, Steinheim, Germany) according to Fitton et al. [37], purified by distillation, and stored under a nitrogen atmosphere over a molecular sieve (3 Å). Polyglycidol (PG) (**1**) was synthesized according to literature [38]. 

Water-sensitive reactions were carried out in a nitrogen atmosphere. Nitrogen (Linde 5.0) was passed over a molecular sieve (4 Å) and finely distributed potassium on aluminum oxide.

### 2.2. Bacteria

To determine the antibacterial activity, polymers were tested against the Gram-negative bacterium *Escherichia coli* (DSM498) and the Gram-positive bacterium *Staphylococcus aureus* (ATCC6538). Overnight cultures of *E. coli* and *S. aureus* with defined bacterial count in Mueller–Hinton broth (Sigma Aldrich Chemie GmbH, Steinheim, Germany, pH = 7.4 ± 0.2) were used as inoculate in the antibacterial test.

### 2.3. Antibacterial Tests of Polymer Solutions

The antibacterial activity of the polymers in solution was determined by measuring the minimal inhibitory concentration (MIC) using the test bacteria mentioned above. Suspensions of strains with known colony forming units (CFU; 1 × 10^6^ ‒2 × 10^6^ CFU·mL^−1^) were incubated at 37 °C in nutrient solutions (Mueller–Hinton Broth, MHB) with different concentrations of the polymer samples. The polymer samples were solubilized in bidistilled water and added to the nutrient solution at a constant ratio of 1:10. The growth of the bacteria was followed during the incubation over 20 h by measuring the optical density at 612 nm every 30 min (with 1400 s of shaking at 100 rpm per 30 min cycle by using a microplate reader/incubator (TECAN Infinite 200 Pro, Tecan Trading AG, Männedorf, Switzerland). The testing is performed with defined concentrations specifically for each polymer until, within the monitoring time of 20 h, no bacterial growth curve is recorded. All experiments were performed in triplicate duplicates and MIC determination was repeated on three different days. The polymers were not sterilized. Sterile controls (defined polymer concentrations in nutrient solution without bacteria) were assessed in every growth curve monitoring testing series. MICs were determined according to broth microdilution in 96-well microtitre plates [39]. Antimicrobial polymer testing reference polymers/controls ε-polylysine and polyhexanide (poly(hexamethylene biguanide)) were used.

The minimal inhibitory concentration (MIC) corresponds to the concentration of the test substance at which a complete inhibition of the growth of the inoculated bacteria was observed by comparison with control samples without test substance. 

### 2.4. Hemolytic Activity

Hemolytic activity was assessed according to literature [40]. Human erythrocytes (from healthy donors, red blood cells (RBC), 0, Rh positive, citrate-stabilized) were obtained by centrifugation (3500 rpm, 12 min) to remove plasma, washed three times in PBS (0.01 M phosphate buffered saline, Sigma Aldrich Chemie GmbH, Steinheim, Germany), and diluted in PBS to obtain a stock solution of 2.5 × 10^8^–3.0 × 10^8^/mL RBC. Solutions of defined polymer concentration (250 µL) were pipetted into 250 μL of the stock solution; the final amount of RBC being 1.2 × 10^8^ –1.5 × 10^8^ RBC/mL. The RBC were exposed for 60 min at 37 °C under 3D-shaking; centrifuged thereafter (4000 rpm, 12 min) and the absorption of the supernatant (diluted 10-fold in PBS) was determined at 414 nm in a microplate reader. As reference solutions, (i) PBS for determining spontaneous hemolysis and (ii) 1% Triton X-100 for 100% hemolysis (positive control) were used. Hemolysis was plotted as a function of polymer concentration and the hemolytic activity was defined as the polymer concentration that causes 50% hemolysis of human RBC relative to the positive control (HC_50_).

### 2.5. Measurements

^1^H NMR and ^13^C NMR were recorded on a Bruker DPX-400 FT-NMR spectrometer (Bruker Corporation, Billerica, MA, USA) at 400 and 101 MHz, respectively. Deuterated chloroform (CDCl_3_), deuterated dimethyl sulfoxide (DMSO-*d*_6_), deuterated acetone (acetone-*d*_6_), and deuterated methanol (MeOD) were used as solvents. The residual solvent signal was used as internal standard. Coupling constants ***J_xy_*** are given in Hz.

Molecular weights (*M*_n,SEC_) and molecular weight distributions (*Đ*) were determined by size exclusion chromatography (SEC). SEC analyses were carried out with DMF as eluent. SEC with DMF (HPLC grade, VWR) as eluent was performed using an Agilent 1100 system (Agilent Technologies, Santa Clara, CA, USA) equipped with a dual RI-/Visco detector (ETA-2020, WGE Dr. Bures GmbH & Co KG, Dallgow-Doeberitz, Germany). The eluent contained 1 g·L^−1^ LiBr (≥99%, Sigma Aldrich Chemie GmbH, Steinheim, Germany). The sample solvent contained traces of distilled water as internal standard. For cationic samples, the eluent contained 2 g·L^−1^ LiBr and 2 g·L^−1^ tris(hydroxymethyl)aminomethane (TRIS, ultrapure grade, ≥99.9%, Sigma Aldrich Chemie GmbH, Steinheim, Germany). One pre-column (8 mm × 50 mm) and four GRAM gel columns (8 mm × 300 mm, Polymer Standards Service, Mainz, Germany) were applied at a flow rate of 1.0 mL·min^−1^ at 40 °C. The diameter of the gel particles measured 10 µm, the nominal pore widths were 30, 100, 1000, and 3000 Å. Calibration was achieved using narrowly distributed poly(methyl methacrylate) standards (Polymer Standards Service, Mainz, Germany). Results were evaluated using the PSS WinGPC UniChrom software (Version 8.1.1, PSS Polymer Standards Service GmbH, Mainz, Germany).

Dialysis was performed in methanol using Biotech CE Tubing (MWCO: 100–500 D, 3.1 mL·cm^−1^, Spectrum Laboratories, Inc., Rancho Dominguez, CA, USA) and Biotech RC Tubing (MWCO: 1 kD, 6.4 mL·cm^−1^, Spectrum Laboratories, Inc., Rancho Dominguez, CA, USA), respectively. The membrane was washed for 15 min in water before use to remove the sodium azide solution.

### 2.6. Synthesis of Poly(glycidyl phenyl carbonate) (P(G^PC^)_27_) (**2**)

PG_27_ (**1**) (2.018 g, 27.24 mmol OH) was dissolved in pyridine (18.87 mL), and a solution of phenyl chloroformate (4.692 g, 29.97 mmol) in dichloromethane (17.5 mL) was added in 30 min at 0 °C using a syringe pump. The reaction mixture was allowed to warm to room temperature and stirred for 20 h. The precipitate was removed by filtration. The solution was washed with water (15 mL), 1 M HCl solution (aq.) (3 × 15 mL), and saturated NaCl solution (aq.) (15 mL). The organic phase was dried over Na_2_SO_4_, filtrated, and the solvent removed under reduced pressure. Polymer **2** was obtained as a brown viscous liquid (3.650 g, 69%). *M*_n,NMR_ = 5243 g·mol^−1^, *M*_n,SEC_ = 4800 g·mol^−1^, *Ð* = 1.14. ^1^H NMR (400 MHz, CDCl_3_) (**2**): δ = 1.79 (m, ArCH_2_*CH_2_*), 2.57 (t, *^3^J_HH_* = 7.8 Hz, Ar*CH_2_*CH_2_), 3.45–3.87 (m, ArCH_2_CH_2_*CH_2_*, O*CH_2_CH*(CH_2_OC=OOPh)O), 4.08–4.42 (m, OCH_2_CH(*CH_2_*OC=OOPh)O), 6.98–7.29 (m, *Ar*CH_2_CH_2_, (OC=OO*Ph*)O) ppm. ^13^C NMR (101 MHz, CDCl_3_) (**2**): δ = 31.2 (ArCH_2_*CH_2_*), 32.3 (Ar*CH_2_*CH_2_), 67.7–69.4 (ArCH_2_CH_2_*CH_2_*, O*CH_2_CH*(CH_2_OC=OOPh)O), 77.4 (OCH_2_CH(*CH_2_*OC=OOPh)O), 121.0 (OC=OO*Ph*)O), 126.1 (*Ar*CH_2_, OC=OO*Ph*)O), 128.4 (*Ar*CH_2_), 128.5 (*Ar*CH_2_), 129.6 (OC=OO*Ph*)O), 141.8 (*Ar*CH_2_), 151.1 (OC=OO*Ph*)O), 153.6 (O*C=O*OPh)O) ppm.

### 2.7. Synthesis of Poly(glycidyl 3-dimethylaminopropylcarbamate-co-glycidyl dodecylcarbamate) (P(G^DMAPA^_15_-co-G^DDA^_12_)) (**3**)

P(G^PC^)_27_ (**2**) (1.509 g, 7.77 mmol carbonate) was dissolved in tetrahydrofuran (15 mL) and a solution of 3-(dimethylamino)-1-propylamine (0.397 g, 3.89 mmol) and dodecylamine (0.721 g, 3.89 mmol) in tetrahydrofuran (15 mL) was added in 1 h at 0 °C using a syringe pump. The reaction was allowed to warm to room temperature and stirred for 42 h. The solvent was removed under reduced pressure and the polymer purified by dialysis in methanol. Polymer **3** was obtained as a yellowish viscous liquid (1.153 g, 62%). *M*_n,NMR_ = 6459 g·mol^−1^, *M*_n,SEC_ = 7600 g·mol^−1^, *Ð* = 1.38. ^1^H NMR (400 MHz, CDCl_3_) (**3**): δ = 0.84 (t, *^3^J_HH_* = 7.0 Hz, NHCH_2_CH_2_(CH_2_)_9_*CH_3_*), 1.21 (s, NHCH_2_CH_2_(*CH_2_*)_9_CH_3_), 1.34–1.51 (m, NHCH_2_*CH_2_*(CH_2_)_9_CH_3_), 1.54–1.68 (m, NHCH_2_*CH_2_*CH_2_N(CH_3_)_2_), 1.77–1.89 (m, ArCH_2_*CH_2_*), 2.16 (s, NHCH_2_CH_2_CH_2_N(*CH_3_*)_2_), 2.27 (t, *^3^J_HH_* = 6.7 Hz, NHCH_2_CH_2_*CH_2_*N(CH_3_)_2_), 2.63 (t, *^3^J_HH_* = 7.6 Hz, Ar*CH_2_*CH_2_), 3.01–3.11 (m, NH*CH_2_*CH_2_(CH_2_)_9_CH_3_), 3.12–3.21 (m, 2H, NH*CH_2_*CH_2_CH_2_N(CH_3_)_2_), 3.45–3.79 (m, ArCH_2_CH_2_*CH_2_*, O*CH_2_CH*(CH_2_OC=ONHR)O), 3.86–4.36 (m, OCH_2_CH(*CH_2_*OC=ONHR)O), 5.88 (br. s, NH), 6.17 (br. s, NH), 7.09–7.25 (m, *Ar*CH_2_CH_2_) ppm. ^13^C NMR (101 MHz, CDCl_3_) (**3**): δ = 14.2 (NHCH_2_CH_2_(CH_2_)_9_*CH_3_*), 22.7 (NHCH_2_(*CH_2_*)_10_CH_3_), 27.0 (NHCH_2_*CH_2_*CH_2_N(CH_3_)_2_, 27.5–32.0 (NHCH_2_(*CH_2_*)_10_CH_3_, Ar*CH_2_CH_2_*), 39.9 (NH*CH_2_*(CH_2_)_10_CH_3_), 41.2 (NH*CH_2_*CH_2_CH_2_N(CH_3_)_2_), 45.5 (NHCH_2_CH_2_CH_2_N(*CH_3_*)_2_), 57.6 (NHCH_2_CH_2_*CH_2_*N(CH_3_)_2_), 64.2–70.0 (ArCH_2_CH_2_*CH_2_*, O*CH_2_CH*(CH_2_OC=ONHR)O), 78.0 (OCH_2_CH(*CH_2_*OC=ONHR)O), 125.8 (*Ar*CH_2_), 128.4 (*Ar*CH_2_), 128.5 (*Ar*CH_2_), 141.9 (*Ar*CH_2_), 156.7 (OCH_2_CH(CH_2_O*C=O*NHR)O) ppm.

### 2.8. Synthesis of Poly(glycidyl 3-aminopropyldiethanolcarbamate-co-glycidyl dodecylcarbamate) (P(G^APDEA^_16_-co-G^DDA^_11_)) (**4**)

P(G^PC^)_27_ (**2**) (0.707 g, 3.51 mmol carbonate) was dissolved in tetrahydrofuran (10 mL) and a solution of *N*-(3-aminopropyl)diethanolamine (0.284 g, 1.75 mmol), and dodecylamine (0.325 g, 1.75 mmol) in tetrahydrofuran (5 mL) was added in 1 h at 0 °C using a syringe pump. The reaction was allowed to warm to room temperature and stirred for 42 h. The solvent was removed under reduced pressure and the polymer purified by dialysis in methanol. Polymer **4** was obtained as a colorless viscous liquid (0.655 g, 66%). *M*_n,NMR_ = 7337 g·mol^−1^, *M*_n,SEC_ = 14,100 g·mol^−1^, *Ð* = 3.56. ^1^H NMR (400 MHz, MeOD) (**4**): δ = 0.92 (t, *^3^J_HH_* = 7.0 Hz, NHCH_2_CH_2_(CH_2_)_9_*CH_3_*), 1.31 (s, NHCH_2_CH_2_(*CH_2_*)_9_CH_3_), 1.44–1.59 (m, NHCH_2_*CH_2_*(CH_2_)_9_CH_3_), 1.62–1.76 (m, NHCH_2_*CH_2_*CH_2_N(CH_2_CH_2_OH)_2_), 1.85–1.92 (m, ArCH_2_*CH_2_*), 2.55–2.74 (m, *CH_2_*N(*CH_2_*CH_2_OH)_2_), 3.06–3.14 (m, NH*CH_2_*CH_2_(CH_2_)_9_CH_3_), 3.15–3.23 (m, NH*CH_2_*CH_2_CH_2_N(CH_2_CH_2_OH)_2_), 3.62 (t, *^3^J_HH_* = 5.4 Hz, CH_2_N(CH_2_*CH_2_*OH)_2_), 3.66–3.81 (m, ArCH_2_CH_2_*CH_2_*, O*CH_2_CH*(CH_2_OC=ONHR)O), 4.00–4.34 (m, OCH_2_CH(*CH_2_*OC=ONHR)O), 7.17–7.32 (m, *Ar*CH_2_CH_2_) ppm. ^13^C NMR (101 MHz, MeOD) (**4**): δ = 14.6 (NHCH_2_CH_2_(CH_2_)_9_*CH_3_*), 23.8–30.9 (NHCH_2_CH_2_(*CH_2_*)_9_CH_3_), 28.2 (NHCH_2_*CH_2_*CH_2_N(CH_2_CH_2_OH)_2_), 33.1 (NHCH_2_*CH_2_*(CH_2_)_9_CH_3_), 40.1 (NH*CH_2_*CH_2_CH_2_N(CH_2_CH_2_OH)_2_), 42.0 (NH*CH_2_*CH_2_(CH_2_)_9_CH_3_), 53.6 (*CH_2_*N(CH_2_CH_2_OH)_2_), 57.6 (CH_2_N(CH_2_*CH_2_*OH)_2_), 60.8 (CH_2_N(*CH_2_*CH_2_OH)_2_), 65.3–79.3 (ArCH_2_CH_2_*CH_2_*, O*CH_2_CH*(*CH_2_*OC=ONHR)O), 129.4 (*Ar*CH_2_), 129.6 (*Ar*CH_2_), 158.7 (OCH_2_CH(CH_2_O*C=O*NHR)O) ppm.

### 2.9. Synthesis of Poly(glycidyl 3-trimethylaminopropylcarbamate-co-glycidyl dodecylcarbamate) (P(G^TMAPA^_15_-co-G^DDA^_12_)) (**5**)

P(G^DMAPA^_15_-*co*-G^DDA^_12_) (**3**) (0.147 g, 0.34 mmol –NMe_2_) was dissolved in THF (3.0 mL). Methyl iodide (0.058 g, 0.41 mmol) was added and the solution was stirred for 20 h at room temperature. Excess methyl iodide and the solvent were removed under reduced pressure and the polymer purified by dialysis in methanol. Polymer **5** was obtained as a yellowish crystalline solid (0.205 g, 89%). *M*_n,NMR_ = 10,111 g·mol^−1^, *M_n_*_,SEC_ = 4400 g·mol^−1^, *Ð* = 1.18. ^1^H NMR (400 MHz, DMSO-*d*_6_) (**5**): δ = 0.74–0.90 (m, NHCH_2_CH_2_(CH_2_)_9_*CH_3_*), 1.20 (s, NHCH_2_CH_2_(*CH_2_*)_9_CH_3_), 1.30–1.44 (m, NHCH_2_*CH_2_*(CH_2_)_9_CH_3_), 1.72–1.91 (m, NHCH_2_*CH_2_*CH_2_N^+^(CH_3_)_3_, ArCH_2_*CH_2_*), 2.55–2.62 (m, *^3^J_HH_* = 7.6 Hz, Ar*CH_2_*CH_2_), 2.85–2.99 (m, NH*CH_2_*CH_2_(CH_2_)_9_CH_3_), 3.00–3.17 (m, NHCH_2_CH_2_CH_2_N^+^(*CH_3_*)_3_, NH*CH_2_*CH_2_CH_2_N^+^(CH_3_)_3_), 3.26–3.43 (m, NHCH_2_CH_2_*CH_2_*N^+^(CH_3_)_3_), 3.44–3.74 (m, ArCH_2_CH_2_*CH_2_*, O*CH_2_CH*(CH_2_OC=ONHR)O), 3.82–4.19 (m, OCH_2_CH(*CH_2_*OC=ONHR)O), 7.10–7.30 (m, *Ar*CH_2_CH_2_) ppm. ^13^C NMR (101 MHz, DMSO-*d*_6_) (**5**): δ = 13.8 (NHCH_2_CH_2_(CH_2_)_9_*CH_3_*), 22.0–28.9 (NHCH_2_(*CH_2_*)_10_CH_3_), 26.2 (NHCH_2_*CH_2_*CH_2_N^+^(CH_3_)_3_), 31.2 (NH*CH_2_*(CH_2_)_10_CH_3_), 37.3 (NH*CH_2_*CH_2_CH_2_N^+^(CH_3_)_3_), 52.2 (NHCH_2_CH_2_CH_2_N^+^(*CH_3_*)_3_), 63.2 (NHCH_2_CH_2_*CH_2_*N^+^(CH_3_)_3_), 68.6–77.0 (ArCH_2_CH_2_*CH_2_*, O*CH_2_CH*(CH_2_OC=ONHR)O), OCH_2_CH(*CH_2_*OC=ONHR)O), 125.5 (*Ar*CH_2_), 128.9 (*Ar*CH_2_), 141.5 (*Ar*CH_2_), 156.0 (OCH_2_CH(CH_2_O*C=O*NHR)O) ppm.

### 2.10. Synthesis of Poly(glycidyl 3-aminopropyldiethanolmethylcarbamate-co-glycidyl dodecylcarbamate) (P(G^APDEMA^_16_-co-G^DDA^_11_)) (**6**) 

P(G^APDEA^_16_-*co*-G^DDA^_11_) (**4**) (0.216 g, 0.47 mmol –NEtOH_2_) was dissolved in THF (2.5 mL). Methyl iodide (0.081 g, 0.57 mmol) was added and the solution was stirred for 20 h at room temperature. Excess methyl iodide and the solvent were removed under reduced pressure and the polymer purified by dialysis in methanol. Polymer **6** was obtained as a slightly yellow solid (0.238 g, 84%). *M*_n,NMR_ = 9608 g·mol^−1^, *M*_n,SEC_ = not measurable. ^1^H NMR (400 MHz, MeOD) (**6**): δ = 0.96 (t, *^3^J_HH_* = 6.3 Hz, NHCH_2_CH_2_(CH_2_)_9_*CH_3_*), 1.35 (s, NHCH_2_CH_2_(*CH_2_*)_9_CH_3_), 1.48–1.65 (m, NHCH_2_*CH_2_*(CH_2_)_9_CH_3_), 1.88–1.99 (m, ArCH_2_*CH_2_*), 2.05–2.23 (m, NHCH_2_*CH_2_*CH_2_N^+^(CH_3_(CH_2_CH_2_OH)_2_)), 2.70–2.78 (m, Ar*CH_2_*CH_2_), 3.09–3.20 (m, NH*CH_2_*CH_2_(CH_2_)_9_CH_3_), 3.24–3.42 (m, NH*CH_2_*CH_2_*CH_2_*N^+^(*CH_3_*(CH_2_CH_2_OH)_2_)), 3.51–3.96 (m, ArCH_2_CH_2_*CH_2_*, O*CH_2_CH*(CH_2_OC=ONHR)O, CH_2_N^+^(CH_3_(*CH_2_*CH_2_OH)_2_)), 4.02–4.42 (m, OCH_2_CH(*CH_2_*OC=ONHR)O, CH_2_N^+^(CH_3_(CH_2_*CH_2_*OH)_2_)), 7.20–7.36 (m, *Ar*CH_2_CH_2_) ppm. ^13^C NMR (101 MHz, MeOD) (**6**): δ = 14.5 (NHCH_2_CH_2_(CH_2_)_9_*CH_3_*), 23.7–31.0 (NHCH_2_CH_2_(*CH_2_*)_9_CH_3_), 28.0 (NHCH_2_*CH_2_*CH_2_N^+^(CH_3_(CH_2_CH_2_OH)_2_), 33.0 (NHCH_2_*CH_2_*(CH_2_)_9_CH_3_), 38.9 (NH*CH_2_*CH_2_CH_2_N^+^(CH_3_(CH_2_CH_2_OH)_2_)), 42.0 (NH*CH_2_*CH_2_(CH_2_)_9_CH_3_), 50.9 (NHCH_2_CH_2_CH_2_N^+^(*CH_3_*(CH_2_CH_2_OH)_2_), 56.8 (NHCH_2_CH_2_*CH_2_*N^+^(CH_3_(CH_2_CH_2_OH)_2_), 62.8–79.1 (ArCH_2_CH_2_*CH_2_*, O*CH_2_CH*(*CH_2_*OC=ONHR)O), 65.4 (CH_2_N^+^(CH_3_(*CH_2_CH_2_*OH)_2_), 129.4 (*Ar*CH_2_), 129.6 (*Ar*CH_2_), 158.7 (OCH_2_CH(CH_2_O*C=O*NHR)O) ppm.

### 2.11. Synthesis of Poly(glycidyl-4-nitrophenyl carbonate) (P(G^NPC^)_27_) (**7**)

PG_27_ (**1**) (2.012 g, 27.16 mmol OH) was dissolved in pyridine (18.85 mL). The 4-nitrophenyl chloroformate (6.023 g, 29.88 mmol) was dissolved in dichloromethane (20 mL) and added to the polymer solution via syringe pump in 30 min at 0 °C. The solution was stirred for 20 h at room temperature. The crude product was washed with water (30 mL), 1 M HCl (aq.) (3 × 30 mL), and saturated NaCl solution (aq.) (30 mL). The organic phase was separated, dried over Na_2_SO_4_ and precipitated in cold MeOH. The solvent was removed under reduced pressure and polymer **7** was obtained as a colorless solid (5.782 g, 89%). *M*_n,NMR_ = 6458 g·mol^−1^, *M*_n,SEC_ = 6600 g·mol^−1^, *Ð* = 1.41. ^1^H NMR (400 MHz, DMSO-*d*_6_) (**7**): δ = 1.69–1.83 (m, ArCH_2_*CH_2_*), 2.54–2.61 (m, Ar*CH_2_*CH_2_), 3.54–3.95 (m, ArCH_2_CH_2_*CH_2_*, O*CH_2_CH*(CH_2_OC=OOArNO_2_)O), 4.15–4.56 (d, *CH_2_*OC=OOArNO_2_), 7.08–7.25 (m, *Ar*CH_2_CH_2_), 7.42 (s, CH_2_OC=OO*Ar*NO_2_), 8.18 (s, CH_2_OC=OO*Ar*NO_2_) ppm. ^13^C NMR (101 MHz, DMSO-*d*_6_) (**7**): δ = 30.8 (ArCH_2_*CH_2_*), 31.6 (Ar*CH_2_*), 68.2 (O*CH_2_*CH(CH_2_OC=OOArNO_2_)O), 76.4 (OCH_2_*CH*(CH_2_OC=OOArNO_2_)O), 76.5 (ArCH_2_CH_2_*CH_2_*), 76.6 (OCH_2_CH(*CH_2_*OC=OOArNO_2_)O), 122.3 (CH_2_OC=OO*Ar*NO_2_), 125.2 (CH_2_OC=OO*Ar*NO_2_), 125.7 (*Ar*CH_2_), 128.2 (*Ar*CH_2_), 128.3 (*Ar*CH_2_), 141.6 (*Ar*CH_2_), 145.0 (CH_2_OC=OO*Ar*NO_2_), 152.0 (*OC=OO*), 155.1 (CH_2_OC=OO*Ar*NO_2_) ppm.

### 2.12. Synthesis of Poly(glycidyl homocysteine thiolactonylcarbamate) (P(G^HCTL^)_27_) (**8**)

P(G^NPC^)_27_ (**7**) (1.020 g, 4.26 mmol carbonate) was dissolved in DMF (10 mL), and 4-(dimethylamino)pyridine (0.053 g, 0.43 mmol) and dl-homocysteine thiolactone hydrochloride (0.655 g, 4.26 mmol) were added. The mixture was cooled to 0 °C and triethylamine (0.862 g, 8.52 mmol) was added over 1 h via syringe pump. The solution was stirred for 20 h at room temperature. DMF was removed under reduced pressure at 50 °C and the crude product was precipitated in MeOH. Drying under reduced pressure at 50 °C gave polymer **8** as a slightly yellow solid (0.694 g, 75%). *M*_n,NMR_ = 5865 g·mol^−1^, *M*_n,SEC_ = 6900 g·mol^−1^, *Ð* = 1.44. ^1^H NMR (400 MHz, DMSO-*d*_6_) (**8**): δ = 1.74–1.83 (m, ArCH_2_*CH_2_*), 2.00–2.48 (m, C=OSCH_2_*CH_2_*CHR), 2.60 (t, *^3^J_H,H_* = 7.5 Hz, Ar*CH_2_*CH_2_), 3.17–3.45 (m, C=OS*CH_2_*CH_2_CHR), 3.47–3.75 (m, ArCH_2_CH_2_*CH_2_*, O*CH_2_CH*(CH_2_OC=ONHR)O), 3.86–4.23 (m, *CH_2_*OC=ONHR), 4.26–4.42 (m, C=OSCH_2_CH_2_*CH*R), 7.14–7.29 (Ar), 7.55 (br s, NH) ppm. ^13^C NMR (101 MHz, DMSO-*d*_6_) (**8**): δ = 26.5 (C=OSCH_2_*CH_2_*CHR), 29.8 (C=OS*CH_2_*CH_2_CHR), 30.9 (ArCH_2_*CH_2_*), 31.6 (Ar*CH_2_*), 59.9 (*CH_2_*OC=ONHR), 63.7, 68.7, 77.2 (ArCH_2_CH_2_*CH_2_*, O*CH_2_CH*(CH_2_OC=ONHR)O), 79.2 (C=OSCH_2_CH_2_*CH*R), 128.3 (*Ar*CH_2_), 128.4 (*Ar*CH_2_), 156.0 (O*C=O*NHR), 205.7 (*C=O*SCH_2_CH_2_CHR) ppm.

### 2.13. Synthesis of P(G^DDAc^)_27_ (**9**)

To a mixture of P(G^HCTL^)_27_ (**8**) (0.301 g, 1.386 mmol HCTL) and dodecyl acrylate (0.833 g, 3.464 mmol) in chloroform (3.0 mL), 3-(dimethylamino)-1-propylamine (0.353 g, 3.464 mmol) was added in 30 min via syringe pump at room temperature. The mixture was stirred for 20 h at room temperature. Chloroform was removed under reduced pressure. Dialysis in acetone gave **9** as a colorless, viscous liquid (0.628 g, 81%). *M*_n,NMR_ = 15,115 g·mol^−1^, *M*_n,SEC_ = 15,300 g·mol^−1^, *Ð* = 1.36. ^1^H NMR (400 MHz, CDCl_3_) (**9**): δ = 0.82 (t, *^3^J_H,H_* = 6.8 Hz, CH_2_CH_2_(CH_2_)_9_*CH_3_*), 1.20 (s, CH_2_CH_2_(*CH_2_*)_9_CH_3_), 1.48–1.69 (m, *CH_2_*CH_2_N(CH_3_)_2_, CH_2_*CH_2_*(CH_2_)_9_CH_3_, ArCH_2_*CH_2_*), 1.76–2.08 (m, CH*CH_2_*CH_2_SCH_2_), 2.15 (s, CH_2_N(*CH_3_*)_2_), 2.22–2.37 (m, *CH_2_*N(CH_3_)_2_), 2.43–2.61 (m, CHCH_2_*CH_2_*SCH_2_*CH_2_*), 2.64–2.78 (m, CHCH_2_CH_2_S*CH_2_*CH_2_), 3.07–3.37 (m, NH*CH_2_*CH_2_CH_2_N(CH_3_)_2_), 3.41–3.75 (m, ArCH_2_CH_2_*CH_2_*, O*CH_2_CH*(CH_2_OC=ONHR)O), 3.77–4.07 (m, *CH_2_*OC=ONH*CH*), 4.10–4.41 (m, O=CO*CH_2_*CH_2_), 7.09–7.23 (m, Ar), 7.76 (br. s, NH) ppm. ^13^C NMR (101 MHz, CDCl_3_) (**9**): δ = 14.1 (CH_2_CH_2_(CH_2_)_9_*CH_3_*), 22.7–31.9 (CH_2_*CH_2_*(*CH_2_*)_9_CH_3_, CH*CH_2_*CH_2_SCH_2_, Ar*CH_2_CH_2_*), 26.8 (CHCH_2_*CH_2_*SCH_2_CH_2_), 28.2 (*CH_2_*CH_2_N(CH_3_)_2_), 28.6 (CHCH_2_CH_2_S*CH_2_*CH_2_), 34.7 (CHCH_2_CH_2_SCH_2_*CH_2_*), 38.7 (NH*CH_2_*CH_2_CH_2_N(CH_3_)_2_), 45.5 (CH_2_N(*CH_3_*)_2_), 54.1 (NH*CH*), 57.9 (*CH_2_*N(CH_3_)_2_), 64.9 (O=CO*CH_2_*CH_2_), 125.9 (*Ar*CH_2_), 128.3 (*Ar*CH_2_), 128.5 (*Ar*CH_2_), 140.0 (*Ar*CH_2_), 156.4 (O*C=O*NH), 171.5 (CH*C=O*NH), 172.0 (CH_2_*C=O*O) ppm.

### 2.14. Synthesis of P(G^DDAc, q^)_27_ (**10**)

P(G^DDAc^)_27_ (**9**) (0.431 g, 0.770 mmol –NMe_2_) was dissolved in THF (9.0 mL), and methyl iodide (0.131 g, 0.923 mmol) was added. The solution was stirred at room temperature for 20 h. Removal of THF and excess methyl iodide under reduced pressure and dialysis in methanol gave polymer **10** as a slightly yellow solid (0.475 g, 88%). *M*_n,NMR_ = 18,947 g·mol^−1^, *M*_n,SEC_ = 11,800 g·mol^−1^, *Ð* = 1.36. ^1^H NMR (400 MHz, CDCl_3_) (**10**): δ = 0.81 (t, *^3^J_H,H_* = 6.5 Hz, CH_2_CH_2_(CH_2_)_9_*CH_3_*), 1.05–1.32 (m, CH_2_CH_2_(*CH_2_*)_9_CH_3_), 1.46–1.62 (m, CH_2_*CH_2_*(CH_2_)_9_CH_3_), 1.85–2.25 (m, *CH_2_*CH_2_N^+^(CH_3_)_3_, ArCH_2_*CH_2_*, CH*CH_2_*CH_2_SCH_2_), 2.39–2.64 (m, CHCH_2_*CH_2_*SCH_2_*CH_2_*), 2.66–2.82 (m, CHCH_2_CH_2_S*CH_2_*CH_2_), 3.05–3.50 (m, NH*CH_2_*CH_2_*CH_2_*N^+^(*CH_3_*)_3_), 3.51–3.83 (m, ArCH_2_CH_2_*CH_2_*, O*CH_2_CH*(CH_2_OC=ONHR)O), 3.84–4.50 (m, *CH_2_*OC=ONH*CH*, O=CO*CH_2_*CH_2_), 7.08–7.24 (m, Ar), 7.77 (br. s, NH) ppm. ^13^C NMR (101 MHz, CDCl_3_) (**10**): δ = 14.1 (CH_2_CH_2_(CH_2_)_9_*CH_3_*), 22.6–31.9 (CH_2_*CH_2_*(*CH_2_*)_9_CH_3_, CH*CH_2_*CH_2_SCH_2_, Ar*CH_2_CH_2_*), 26.7 (CHCH_2_*CH_2_*SCH_2_CH_2_), 28.3 (*CH_2_*CH_2_N^+^(CH_3_)_3_), 28.6 (CHCH_2_CH_2_S*CH_2_*CH_2_), 34.8 (CHCH_2_CH_2_SCH_2_*CH_2_*), 40.1 (NH*CH_2_*CH_2_CH_2_N^+^(CH_3_)_3_), 53.8 (CH_2_N^+^(*CH_3_*)_3_, NH*CH*), 64.9 (*CH_2_*N^+^(CH_3_)_3_, O=CO*CH_2_*CH_2_), 156.5 (O*C=O*NH), 172.1 (CH*C=O*NH), 172.8 (CH_2_*C=O*O) ppm.

### 2.15. Synthesis of P(G^DMAPA^_14_-co-G^DDADDAc^_13_) (**11**)

P(G^NPC^)_27_ (**7**) (0.541 g, 2.26 mmol carbonate) was dissolved in DMF (11 mL) and 3-(dimethylamino)-1-propylamine (0.115 g, 1.13 mmol) was added in 30 min via syringe pump. The mixture was stirred for 20 h at room temperature. dl-homocysteine thiolactone hydrochloride (0.174 g, 1.13 mmol) and 4-(dimethylamino)pyridine (0.013 g, 0.11 mmol) were added. The mixture was cooled to 0 °C, and triethylamine (0.229 g, 2.26 mmol) was added over 1 h via syringe pump. The solution was stirred for 20 h at room temperature. DMF was removed under reduced pressure at room temperature. The crude product was dissolved in chloroform, and dodecyl acrylate (0.680 g, 2.83 mmol) was added. The mixture was cooled to 0 °C, and dodecylamine (0.525 g, 2.83 mmol) was added in 30 min using a syringe pump. After stirring for 20 h at room temperature, the solvent was removed and the crude product was purified by dialysis in acetone. Polymer **11** was received as a slightly yellow, viscous liquid (0.469 g, 48%). *M*_n,NMR_ = 11,631 g·mol^−1^, *M*_n,SEC_ = 10,800 g·mol^−1^, *Ð* = 1.36. ^1^H NMR (400 MHz, CDCl_3_/acetone-*d*_6_ (6:4)) (**11**): δ = 0.81 (t, *^3^J_H,H_* = 6.6 Hz CH_2_CH_2_(CH_2_)_9_*CH_3_*), 1.20 (s, CH_2_CH_2_(*CH_2_*)_9_CH_3_), 1.38–1.50 (m, NHCH_2_*CH_2_*(CH_2_)_9_CH_3_), 1.51–1.64 (m, OCH_2_*CH_2_*(CH_2_)_9_CH_3_), 1.70–1.86 (m, *CH_2_*CH_2_N(CH_3_)_2_), 1.88–2.01 (m, CH*CH_2_*CH_2_SCH_2_), 2.29–2.77 (m, *CH_2_*N(*CH_3_*)_2_, CHCH_2_*CH_2_*S*CH_2_CH_2_*), 2.97–3.30 (m, NH*CH_2_*CH_2_CH_2_N(CH_3_)_2_, NH*CH_2_*CH_2_(CH_2_)_9_CH_3_), 3.44–3.79 (m, ArCH_2_CH_2_*CH_2_*, O*CH_2_CH*(CH_2_OC=ONHR)O), 3.90–4.34 (m, *CH_2_*OC=ONHR, *CH_2_*OC=ONH*CH*, O=CO*CH_2_*CH_2_), 7.07–7.24 (m, Ar) ppm. ^13^C NMR (101 MHz, CDCl_3_/acetone-*d*_6_ (6:4)) (**11**): δ = 13.5 (CH_2_CH_2_(CH_2_)_9_*CH_3_*), 22.1–31.4 (CH_2_*CH_2_*(*CH_2_*)_9_CH_3_, CH*CH_2_CH_2_*S*CH_2_*, Ar*CH_2_CH_2_*, *CH_2_*CH_2_N(CH_3_)_2_), 34.2 (CHCH_2_CH_2_SCH_2_*CH_2_*), 38.4 (NH*CH_2_*CH_2_CH_2_N(CH_3_)_2_), 39.0 (NH*CH_2_*CH_2_(CH_2_)_9_CH_3_), 43.7 (CH_2_N(*CH_3_*)_2_), 53.3 (NH*CH*), 56.0 (*CH_2_*N(CH_3_)_2_), 64.2 (O=CO*CH_2_*CH_2_), 127.8 (*Ar*CH_2_), 127.9 (*Ar*CH_2_), 155.9 (O*C=O*NH), 156.2 (O*C=O*NH), 171.4 (CH*C=O*NH), 172.1 (CH_2_*C=O*O) ppm.

### 2.16. Synthesis of P(G^TMAPA^_14_-co-G^DDADDAc^_13_) (**12**)

P(G^DMAPA^_14_-*co*-G^DDADDAc^_13_) (**11**) (0.179 g, 0.20 mmol –NMe_2_) was dissolved in THF (2.0 mL), and methyl iodide (0.036 g, 0.25 mmol) was added. The solution was stirred at room temperature for 20 h. Removal of THF and excess methyl iodide under reduced pressure and dialysis in methanol gave polymer **12** as a slightly yellow solid (0.203 g, quant.). *M*_n,NMR_ = 13,476 g·mol^−1^, *M*_n,SEC_ = 9400 g·mol^−1^, *Ð* = 1.41. ^1^H-NMR (400 MHz, CDCl_3_/acetone-*d*_6_ (6:4)) (**12**): δ = 0.80 (t, *^3^J_H,H_* = 6.8 Hz CH_2_CH_2_(CH_2_)_9_*CH_3_*), 1.18 (s, CH_2_CH_2_(*CH_2_*)_9_CH_3_), 1.36–1.48 (m, NHCH_2_*CH_2_*(CH_2_)_9_CH_3_), 1.50–1.66 (m, OCH_2_*CH_2_*(CH_2_)_9_CH_3_), 1.74–2.01 (m, *CH_2_*CH_2_N^+^(CH_3_)_3_, CH*CH_2_*CH_2_SCH_2_), 2.42–2.89 (m, CHCH_2_*CH_2_*S*CH_2_CH_2_*), 2.94–3.45 (m, NH*CH_2_*CH_2_*CH_2_*N^+^(*CH_3_*)_3_, NH*CH_2_*CH_2_(CH_2_)_9_CH_3_), 3.49–3.81 (m, ArCH_2_CH_2_*CH_2_*, O*CH_2_CH*(CH_2_OC=ONHR)O), 3.87–4.38 (m, *CH_2_*OC=ONHR, *CH_2_*OC=ONH*CH*, O=CO*CH_2_*CH_2_), 7.03–7.23 (m, Ar) ppm. ^13^C NMR (101 MHz, CDCl_3_/acetone-*d*_6_ (6:4)) (**12**): δ = 13.4 (CH_2_CH_2_(CH_2_)_9_*CH_3_*), 22.1–31.3 (CH_2_*CH_2_*(*CH_2_*)_9_CH_3_, CH*CH_2_CH_2_*S*CH_2_*, Ar*CH_2_CH_2_*, *CH_2_*CH_2_N(CH_3_)_2_), 34.2 (CHCH_2_CH_2_SCH_2_*CH_2_*), 38.9 (NH*CH_2_*CH_2_(CH_2_)_9_CH_3_), 43.0 (NH*CH_2_*CH_2_CH_2_N^+^(CH_3_)_2_), 53.0 (CH_2_N^+^(*CH_3_*)_3_), 53.8 (NH*CH*), 64.1 (*CH_2_*N(CH_3_)_2_,2 O=CO*CH_2_*CH_2_), 127.8 (*Ar*CH_2_), 127.9 (*Ar*CH_2_), 141.3 (*Ar*CH_2_), 155.9 (O*C=O*NH), 156.4 (O*C=O*NH), 171.3 (CH*C=O*NH), 171.4 (CH_2_*C=O*O) ppm.

## 3. Results and Discussion

In the current work, we present the synthesis and characterization of various novel cationic–hydrophobic functionalized polyglycidols. The functional polyethers are evaluated in regard to their antibacterial activity against *E. coli* and *S. aureus*, induced by different microstructures.

**Figure 1:**
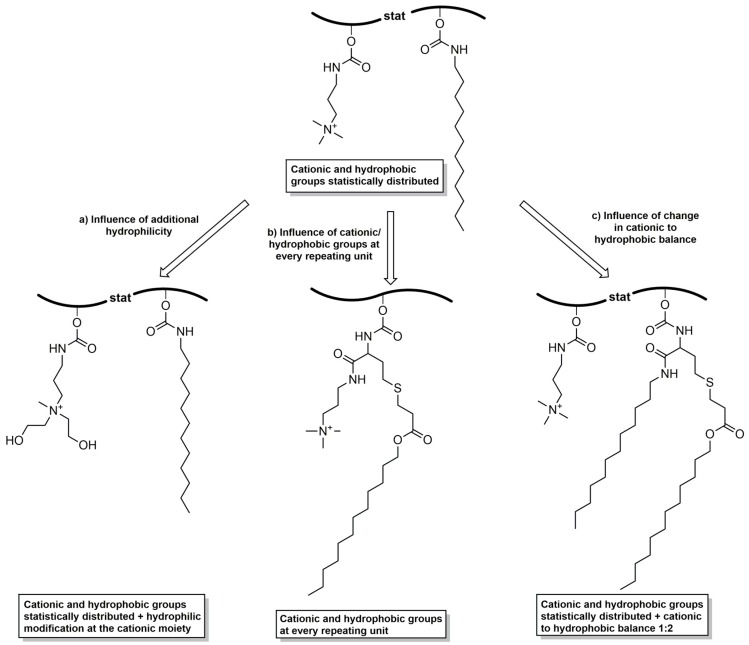
Comparison of various cationic–hydrophobic functionalized polyglycidols in regard to their antibacterial activity against *E. coli* and *S. aureus* to examine the structure–property relationship.

Thereto, a polyglycidol with statistically distributed cationic and hydrophobic groups (cationic–hydrophobic ratio of 1:1) was compared to (a) a polyglycidol with a hydrophilic modification at the cationic moieties; (b) a polyglycidol with cationic and hydrophobic functionalities at every repeating unit; and (c) a polyglycidol with a cationic–hydrophobic balance of 1:2 (Figure 1).

Linear polyglycidol was synthesized by anionic ring-opening polymerization of ethoxyethyl glycidyl ether with 3-phenyl-1-propanol as initiator, followed by removal of the acetal protecting groups under acidic conditions [38]. Polyglycidol with 27 repeating units (PG_27_ (**1**)) and *M*_n,SEC_ = 2900 g·mol^−1^ was obtained with a narrow molecular weight distribution (*Ð* = 1.13). The ^1^H, ^13^C NMR spectra and SEC analysis of PG_27_ (**1**) can be found in the supporting information (Figures S1–S3).

### 3.1. Synthesis of P(G^TMAPA^_15_-co-G^DDA^_12_) (**5**) and P(G^APDEMA^_16_-co-G^DDA^_11_) (**6**)

The general approach for the functionalization of linear polyglycidol with cationic and hydrophobic groups starts with the introduction of active ester functionalities to the polymer backbone. The active esters allow the reaction with primary amines under the selective formation of carbamate moieties. Quaternization of introduced tertiary amines gave the cationic component.

**Scheme 1:**
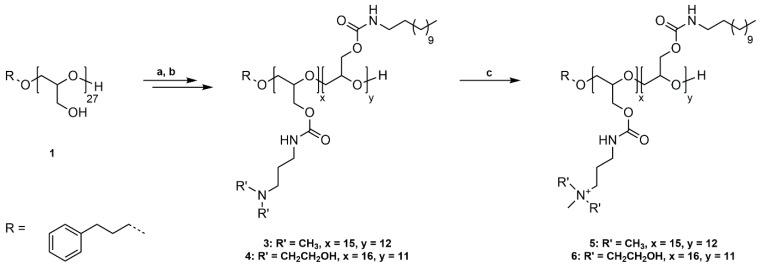
Synthetic pathway to P(G^TMAPA^_15_-*co*-G^DDA^_12_) (**5**) and P(G^APDEMA^_16_-*co*-G^DDA^_11_) (**6**). (**a**) Functionalization of PG_27_ (**1**) with phenyl chloroformate, pyridine/DCM, rt, 20 h; (**b**) Reaction of P(G^PC^)_27_ (**2**) with DDA and DMAPA/APDEA, THF, rt, 42 h; (**c**) Quaternization of tertiary amines with methyl iodide, THF, rt, 20 h.

PG_27_ (**1**) was reacted with phenyl chloroformate in pyridine/dichloromethane at room temperature (Scheme 1a). For purification, poly(glycidyl phenyl carbonate)_27_ (**2**) was washed with water, 1 M HCl solution (aq.), and saturated NaCl solution (aq.) to remove pyridine hydrochloride and excess pyridine. The successful functionalization was confirmed by ^1^H NMR, ^13^C NMR spectroscopy, and SEC analysis (Figures S4–S6). The introduced phenyl carbonate groups are excellent electrophiles for the substitution reaction with non-functionalized, primary amines. P(G^PC^)_27_ (**2**) was subsequently reacted with dodecylamine (DDA) and 3-(dimethylamino)-1-propylamine (DMAPA) or *N*-(3-aminopropyl)diethanolamine (APDEA) in THF at room temperature in a 1:1 ratio (Scheme 1b). The prepared poly(glycidyl 3-dimethylaminopropylcarbamate-*co*-glycidyl dodecylcarbamate) (P(G^DMAPA^_15_-*co*-G^DDA^_12_)) (**3**) and poly(glycidyl 3-aminopropyldiethanolcarbamate-*co*-glycidyl dodecylcarbamate) (P(G^APDEA^_16_-*co*-G^DDA^_11_)) (**4**) were purified by dialysis in methanol and characterized by ^1^H NMR, ^13^C NMR spectroscopy, and SEC analysis (Figures S7–S12). In both cases, the different functional groups are statistically distributed along the polyglycidol backbone. However, the higher reactivity of DMAPA and APDEA in comparison to DDA leads to a slightly uneven ratio of functionalities. P(G^DMAPA^_15_-*co*-G^DDA^_12_) (**3**) and P(G^APDEA^_16_-*co*-G^DDA^_11_) (**4**) were further reacted with an excess of methyl iodide in THF at room temperature to quaternize the tertiary amine moieties (Scheme 1c). The synthesized poly(glycidyl 3-trimethylaminopropylcarbamate-*co*-glycidyl dodecylcarbamate) (P(G^TMAPA^_15_-*co*-G^DDA^_12_)) (**5**) and poly(glycidyl 3-aminopropyldiethanolmethylcarbamate-*co*-glycidyl dodecylcarbamate) (P(G^APDEMA^_16_-*co*-G^DDA^_11_)) (**6**) were purified by dialysis in methanol and analyzed by ^1^H NMR, ^13^C NMR, and SEC analysis.

**Figure 2:**
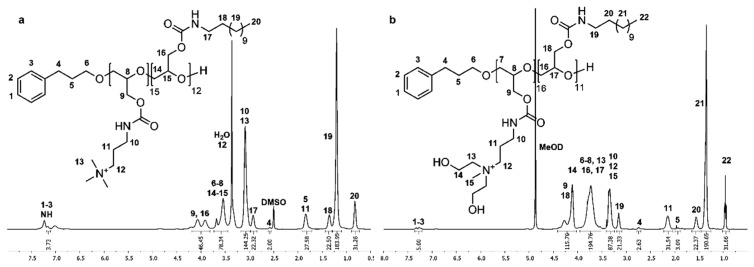
^1^H NMR spectra of P(G^TMAPA^_15_-*co*-G^DDA^_12_) (**5**) measured in DMSO-*d*_6_ (**a**) and P(G^APDEMA^_16_-*co*-G^DDA^_11_) (**6**) measured in MeOD (**b**).

^1^H and ^13^C NMR spectra of P(G^TMAPA^_15_-*co*-G^DDA^_12_) (**5**) in DMSO-*d*_6_ show characteristic signals of the dodecyl and trimethylpropylammonium groups adjacent to the carbamate moieties, proving the successful functionalization of PG_27_ (**1**). In the ^1^H NMR spectrum (Figure 2a), the characteristic signals of the dodecyl functionality appear as three multiplets at δ = 0.74–0.90 (Signal 20), δ = 1.30–1.44 (Signal 18), and δ = 2.85–2.99 ppm (Signal 17), and a singlet at δ = 1.20 ppm (Signal 19). The trimethylpropylammonium groups are shown as three multiplets δ = 1.72–1.91 (Signal 11), δ = 3.00–3.17 (Signal 10/13), and δ = 3.26–3.43 ppm (Signal 12). A multiplet at δ = 3.82–4.19 ppm (Signal 9/16) shows the methylene group of the glycidol repeating unit. In the ^13^C NMR spectrum (Figure S13), the distinctive signals of the dodecyl groups are found at δ = 13.8 (Signal 23), δ = 22.0–28.9 (Signal 22), and δ = 31.2 ppm (Signal 21). The representative signals of the trimethylpropylammonium functionality are shown at δ = 26.2 (Signal 13), δ = 37.3 (Signal 12), δ = 52.2 (Signal 15), and δ = 63.2 ppm (Signal 14). Further, the signal of the carbamate groups can be found at δ = 156.0 ppm (Signal 11/19).

The number of DMAPA and DDA groups attached to PG_27_ (**1**) was calculated by comparing the signal intensity of one methylene group of the 3-phenyl-1-propanol (Signal 4) used in the synthesis of **1** with signals 10/11/13 and signals 18–20, respectively. The absolute molecular weight (M_n,NMR_) was calculated using the 3-phenyl-1-propyl end group as an internal reference (*M*_n,NMR_ = 10,111 g·mol^−1^). Full functionalization of PG_27_ (**1**) was reached.

SEC analysis using DMF as eluent confirms the synthesis of P(G^TMAPA^_15_-*co*-G^DDA^_12_) (**5**) with *M*_n,SEC_ = 4400 g·mol^−1^ and a molecular weight distribution of *Ð* = 1.18 (Figure S14). However, due to the amphiphilic nature of this polymer and resulting interactions with the column, the molecular weight measured by SEC analysis is lower than the molecular weight calculated from the ^1^H NMR spectrum.

^1^H and ^13^C NMR spectra of P(G^APDEMA^_16_-*co*-G^DDA^_11_) (**6**) were measured in MeOD due to the poor solubility of the polymer in DMSO-*d*_6_. In the ^1^H NMR spectrum (Figure 2b), this leads to a shift of all signals to lower field. Thus, the characteristic signals for the dodecyl functionality and the cationic moiety can be found in analogy to P(G^TMAPA^_15_-*co*-G^DDA^_12_) (**5**). Additionally, the signals of the hydroxyethyl group can be found in the multiplets at δ = 3.51–3.96 (Signal 13) and δ = 4.02–4.42 ppm (Signal 14). In the ^13^C NMR spectrum (Figure S15), the signals are also shifted to lower fields in comparison with P(G^TMAPA^_15_-*co*-G^DDA^_12_) (**5**), except for signals of the methyl group and methylene group adjacent to the cationic moiety that are shifted to higher fields. The methyl group can be found at δ = 50.9 ppm (Signal 17) and the methylene group is found at δ = 56.8 ppm (Signal 14). Additionally, the hydroxyethyl group is shown at δ = 65.4 ppm (Signal 15/16). The number of functionalities and the absolute molecular weight was calculated as described previously (M_n.NMR_ = 9608 g·mol^−1^). Both spectra confirm the successful synthesis of P(G^APDEMA^_16_-*co*-G^DDA^_11_) (**6**).

SEC analysis of P(G^APDEMA^_16_-*co*-G^DDA^_11_) (**6**) using DMF was not possible. SEC analysis of the precursor P(G^APDEA^_16_-*co*-G^DDA^_11_) (**4**) confirmed the successful synthesis with *M*_n,SEC_ = 14,100 g·mol^−1^ and a molecular weight distribution of *Ð* = 3.56 (Figure S12). However, hydrogen bonding between polymer molecules leads to the generation of polymer aggregates and, thus, a high molecular weight and a broad distribution. 

### 3.2. Synthesis of P(G^DDAc, q^)_27_ (**10**)

The previous part showed that phenyl carbonates are excellent electrophiles for the substitution reaction of non-functionalized, reactive, primary amines. For the reaction with homocysteine thiolactone the electrophilicity of the carbonate needs to be higher than that of the carbonyl group of the thiolactone ring to prevent the reaction of the homocysteine thiolactone with itself. We have shown in our previous work that 4-nitrophenyl carbonates show a higher reactivity compared to phenyl carbonates in aminolysis reactions and are suitable for the reaction with functional, primary amines [41,42].

**Scheme 2:**
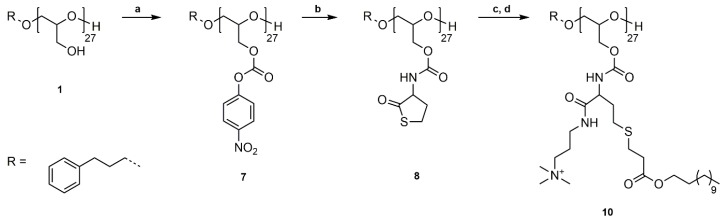
Synthetic pathway to P(G^DDAc, q^)_27_ (**10**). (**a**) Functionalization of PG_27_ (**1**) with 4-nitrophenyl chloroformate, pyridine/DCM, rt, 20 h; (**b**) Reaction of P(G^NPC^)_27_ (**7**) with dl-homocysteine thiolactone hydrochloride, 4-DMAP, Et_3_N, DMF, rt, 20 h; (**c**) Ring-opening reaction with DMAPA, followed by thiol-ene reaction with dodecyl acrylate, CHCl_3_, rt, 20 h; (**d**) Quaternization of tertiary amines with methyl iodide, THF, rt, 20 h.

PG_27_ (**1**) was reacted with 4-nitrophenyl chloroformate in pyridine/dichloromethane at room temperature (Scheme 2a). For purification, poly(glycidyl 4-nitrophenyl carbonate)_27_ (**7**) was washed with water, 1 M HCl solution (aq.), and saturated NaCl solution (aq.) to remove pyridine hydrochloride and excess pyridine, and precipitated in methanol. The successful functionalization was confirmed by ^1^H, ^13^C NMR spectroscopy, and SEC analysis (Figures S16–S18). P(G^NPC^)_27_ was afterwards reacted with dl-homocysteine thiolactone hydrochloride in DMF at room temperature using catalytic amounts of 4-DMAP and Et_3_N as a base (Scheme 2b). The resulting poly(glycidyl homocysteine thiolactonylcarbamate)_27_ (P(G^HCTL^)_27_) (**8**) was purified by precipitation in methanol and characterized by ^1^H, ^13^C NMR spectroscopy, and SEC analysis (Figures S19–S21). In a one-pot reaction, P(G^HCTL^)_27_ (**8**) was reacted with DMAPA under ring-opening in CHCl_3_ at room temperature, and the generated thiol was converted with dodecyl acrylate in a thiol-ene reaction. The synthesized P(G^DDAc^)_27_ (**9**) was purified by dialysis in acetone and characterized by ^1^H NMR, ^13^C NMR spectroscopy, and SEC analysis (Figures S22–S24). P(G^DDAc^)_27_ (**9**) was subsequently quaternized with methyl iodide in THF at room temperature. The crude product was dialyzed in methanol to remove excess methyl iodide, and P(G^DDAc, q^) (**10**) was described by ^1^H, ^13^C NMR spectroscopy, and SEC analysis. 

**Figure 3:**
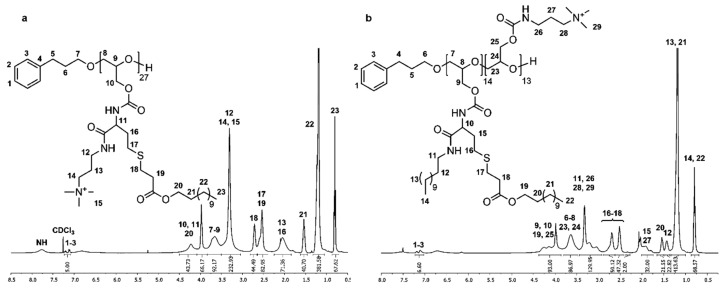
^1^H NMR spectra of P(G^DDAc, q^)_27_ (**10**) measured in CDCl_3_ (**a**) and P(G^TMAPA^_14_-*co*-G^DDADDAc^_13_) (**12**) measured in CDCl_3_/acetone (6:4) (**b**).

P(G^DDAc, q^)_27_ (**10**) exhibits characteristic signals of the introduced functionalities in the ^1^H and ^13^C NMR spectra measured in DMSO-*d*_6_. In the ^1^H NMR spectrum (Figure 3a), the trimethylpropylammonium group is described by two multiplets at δ = 1.85–2.25 (Signal 13) and δ = 3.05–3.50 ppm (Signals 12/14/15). The dodecyl acrylate moiety is shown as a triplet at δ = 0.81 ppm (Signal 23) and two multiplets at δ = 1.05–1.32 (Signal 22) and δ = 1.46–1.62 ppm (Signal 21). Two multiplets at δ = 2.39–2.64 (Signals 17/19) and δ = 2.66–2.82 ppm (Signal 18) are characteristic for the methylene groups adjacent to the thioether. The signals at δ = 3.84–4.50 ppm (Signals 10/11/20) show the methylene groups adjacent to the ester functionality and the single proton adjacent to the carbamate moiety. The multiplet also contains the methylene groups of the polyglycidol repeating unit adjacent to the carbamate. In the ^13^C NMR spectrum (Figure S25), the distinctive signals can be found at δ = 28.3 (Signal 15), δ = 40.1 (Signal 14), δ = 53.8 (Signal 17), and δ = 64.9 ppm (Signal 16) for the trimethylpropylammonium functionality; at δ = 14.1 (Signal 26), δ = 22.6–31.9 (Signals 24/25), and δ = 64.9 ppm (Signal 23) for the dodecyl acrylate; and at δ = 26.7, δ = 28.6 (Signal 18–20) and δ = 34.8 ppm (Signal 21) for the methylene groups adjacent to the thioether. The number of functionalities and the absolute molecular weight was calculated as described previously (*M*_n.NMR_ = 18,947 g·mol^−1^). Both spectra confirm the successful synthesis of P(G^DDAc, q^) (**10**).

SEC analysis using DMF as eluent also confirms the successful synthesis of P(G^DDAc, q^)_27_ (**10**) with *M*_n,SEC_ = 11,800 g·mol^−1^ and a molecular weight distribution of *Ð* = 1.36 (Figure S26).

### 3.3. Synthesis of P(G^TMAPA^_14_-co-G^DDADDAc^_13_) (**12**)

**Scheme 3:**
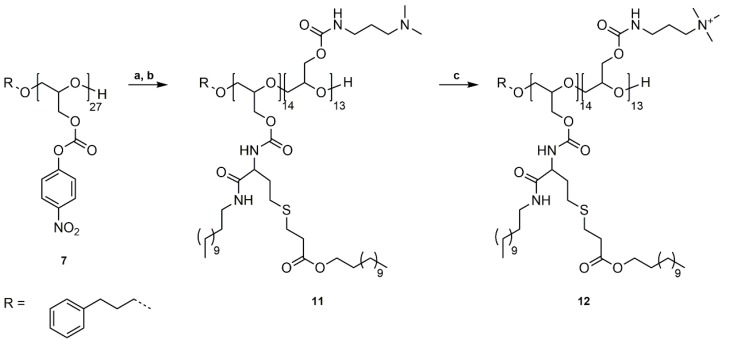
Synthetic pathway to P(G^TMAPA^_14_-*co*-G^DDADDAc^_13_) (**12**). (**a**) (I) Reaction of P(G^NPC^)_27_ (**7**) with DMAPA, DMF, rt, 20 h, (II) Reaction with dl-homocysteine thiolactone hydrochloride, 4-DMAP, Et_3_N, DMF, 20 h; (**b**) Ring-opening reaction with dodecylamine, followed by thiol-ene reaction with dodecyl acrylate, CHCl_3_, rt, 20 h; (**c**) Quaternization of tertiary amines with methyl iodide, THF, rt, 20 h.

The preparation of P(G^TMAPA^_14_-*co*-G^DDADDAc^_13_) (**12**) combines both previously described synthetic approaches. In a one-pot synthesis, P(G^NPC^)_27_ (**7**) was first reacted with DMAPA in DMF at room temperature for 20 h (Scheme 3a). After full conversion of the primary amine (monitored by ^1^H NMR spectroscopy), 4-DMAP, dl-homocysteine thiolactone hydrochloride, and triethylamine were added, and the reaction was stirred for another 20 h at room temperature. The crude product was reacted without further purification with dodecylamine and dodecyl acrylate in chloroform at room temperature (Scheme 3b). The crude product was purified by dialysis in acetone and P(G^DMAPA^_14_-*co*-G^DDADDAc^_13_) (**11**) characterized by ^1^H NMR, ^13^C NMR spectroscopy, and SEC analysis (Figures S27–S29).

P(G^DMAPA^_14_-*co*-G^DDADDAc^_13_ (**11**) was subsequently quaternized with methyl iodide in THF at room temperature, purified by dialysis in methanol, and characterized by the previously described methods (Scheme 3c).

Due to the strong amphiphilic nature of P(G^TMAPA^_14_-*co*-G^DDADDAc^_13_) (**12**), ^1^H and ^13^C NMR spectroscopy were performed in a mixture of CDCl_3_ and acetone-*d*_6_. In the ^1^H NMR spectrum (Figure 3b), the distinctive signals of the hydrophobic moiety are found at δ = 0.80 (Signal 14/22), δ = 1.18 (Signal 13/21), δ = 1.36–1.48 (Signal 12), and δ = 1.50–1.66 ppm (Signal 20). The methylene group adjacent to the ester is shown at δ = 3.87–4.38 ppm (Signal 19). This signal also includes the methylene groups (Signal 9/25) and the single proton (Signal 10) adjacent to the carbamate moieties. Specific signals of the cationic functionalities are found at δ = 1.74–2.01 (Signal 27) and δ = 2.94–3.45 ppm (Signal 26/28/29). The shift of the signals of all functional groups is in good comparison with the polymers described earlier. In the ^13^C NMR spectrum, the shifts of the various signals are also in good comparison to the previously characterized cationic–hydrophobic polymers (Figure S30). Both spectra confirm the successful synthesis of P(G^TMAPA^_14_-*co*-G^DDADDAc^_13_) (**12**). 

The number of functionalities and the absolute molecular weight was calculated as described previously (*M*_n.NMR_ = 13,476 g·mol^−1^). Although, the integral values shown in the ^1^H NMR spectra are too low to be accurate for a polyether with 27 repeating units, the ratio of hydrophobic to cationic moieties is accepted as accurate. The absolute values of hydrophobic to cationic groups is extrapolated to match the expected number of repeating units. Fractionation towards lower molecular weights is excluded based on the method of purification. 

SEC analysis using DMF as eluent also confirms the successful synthesis of P(G^TMAPA^_14_-*co*-G^DDADDAc^_13_) (**12**) with *M*_n,SEC_ = 9400 g·mol^−1^ and a molecular weight distribution of *Ð* = 1.41 (Figure S31).

### 3.4. Antibacterial Efficacy

To assess the influence of the microstructure of the functional polyglycidols on their antibacterial efficacy, the minimal inhibitory concentrations of the four synthesized polymers against *E. coli* and *S. aureus* were determined (Table 1).

In comparison to the other three polymers (**6**, **10**, and **12**) P(G^TMAPA^_15_-*co*-G^DDA^_12_) (**5**)—with cationic and hydrophobic groups statistically distributed at the polyglycidol backbone—exhibited the lowest MIC against both *E. coli* and *S. aureus*. A hydrophilic functionalization at the quaternary amine (P(G^APDEMA^_16_-*co*-G^DDA^_11_) (**6**)) does not affect the efficacy against *E. coli*, but slightly impairs the efficacy against *S. aureus*. 

When the cationic and the hydrophobic residues are located at every repeating unit of the polymer and are connected by a short spacer (P(G^DDAc, q^)_27_ (**10**)), the efficacy against *E. coli* remains the same as for **5** and **6**. However, the efficacy against *S. aureus* decreases significantly by a factor of 10. The molecular weight of polymer **10** is approximately twice as high as the molecular weight of polymers **5** and **6**. Thus, the higher MIC values in the case of *S. aureus* compared to *E. coli* might result from a sieving effect by the thick peptidoglycan layer of the Gram-positive bacterium. This was also discussed by Lienkamp et al. [43]. 

In polymer **10**, the hydrophobic and cationic residues are connected by a spacer and are not directly linked to each other. Opposite to the resulting antibacterial effect of the latter (polymer **10**), functionalized branched poly(ethylene imine)s (PEI) with amphiphilic grafts had better antibacterial properties against *S. aureus* than PEI with randomly linked cationic and hydrophobic grafts. However, best results were obtained with the cationic residue directly linked to the aliphatic residue without a spacer in between [30]. Thus, an influence of the kind of polymer backbone on the polymer architecture and of the kind of amphiphilic functionality on the antibacterial effect can be discussed.

Enhancing the hydrophobic share in comparison to the cationic functionality (P(G^TMAPA^_14_-*co*-G^DDADDAc^_13_) (**12**) with two hydrophobic residues in the same polymer segment, connected by a spacer) leads to a decrease of efficacy by a factor of 10 for both *E. coli* and *S. aureus*. The high amount of hydrophobic residues induces aggregation in aqueous media, which might lead to a partial unavailability of functional groups of the polymer. Thus, interaction with the bacterial cell envelope is impaired, since a certain amount of cationic charge is necessary to initiate the binding to the bacterial cell envelope as the first step in the cascade of amphipathic polymers’ mode of action.

Compared to the 1:2 ratio of cationic to hydrophobic residues in polymer **12**, branched PEI with functionalities introduced via carbonate coupler chemistry and a higher ratio of cationic to hydrophobic residues resulted in higher efficacies in the case of *E. coli* [29]. Furthermore, azetidinium functionalized poly(vinylamine)s with a higher ratio of hydrophobic to cationic moieties resulted in better antibacterial efficacies against *E. coli* and against *S. aureus* [44]. Thus, apparently there is a strong influence of the polymer backbone and the structure of the cationic and hydrophobic side chains on the microstructure and the efficacy besides the hydrophilic to lipophilic balance of the respective polymer. 

Additionally, polymers functionalized with amphiphilic couplers where the cationic moiety was directly linked to the hydrophobic residue exhibited the lowest MIC compared to randomly distributed cationic and hydrophobic groups linked via a spacer. Ganewatta et al. refer to a “same-centered” repeat unit structure with the hydrophobic moiety being directly accompanied by the charged moiety, i.e., the functional groups not being spatially separated over the backbone, leading to an amphiphilic balance at the monomer level [45].

### 3.5. Hemolytic Activity 

To understand the selectivity of polymers **5**, **6**, **10**, and **12** to affect bacterial cells and mammalian cells, a hemolysis test was performed, and the concentration required for 50% lysis of human red blood cells RBCs was determined (HC_50_, Table 1). Linear polyglycidol PG_27_ (**1**) did not show any significant lysis of RBCs even at the highest concentration (500 μg/mL) tested. For polymer **5** at the concentrations tested (10–500 µg/mL), no HC_50_ value could be given at the tested concentrations (10–500 µg/mL). Hemolysis amounted to about 6% at 10 µg/mL, 30% at 30 µg/mL, and 40% at 500 µg/mL. However, agglutination of RBCs occurred at tested concentrations. HC_50_ value for polymer **6** amounted to 100 μg/mL. The higher value of HC_50_ compared to the MIC_100_ against *E. coli* and *S. aureus* (30 µg/mL) proved that polymer **6** has a selectivity to differentiate between mammalian cells and bacterial cell walls. For comparison, the HC_50_ values of polymers **10** and **12** were below the lowest concentration (10 µg/mL) tested, i.e., had a low selectivity for bacterial cell envelopes. 

## 4. Conclusions

We successfully synthesized various novel cationic–hydrophobic functionalized polyglycidols. A polyglycidol with statistically distributed cationic and hydrophobic groups (cationic–hydrophobic ratio of 1:1), a polyglycidol with a hydrophilic modification at the cationic moieties, a polyglycidol with cationic and hydrophobic functionalities at every repeating unit, and a polyglycidol with a cationic–hydrophobic balance of 1:2 were characterized by ^1^H NMR, ^13^C NMR spectroscopy, and SEC analyses and evaluated in regard to their antibacterial activity.

Antibacterial polyglycidols with statistic distribution of cationic and hydrophobic residues are equally active against a Gram-negative and a Gram-positive bacterial strain. In contrast to this, the efficacy of amphipathic poly(ethylene imine)s is higher by a factor of 10 against Gram-positive bacteria than against Gram-negative ones. However, antibacterial polyglycidols are equally or better active against *E. coli* than against *S. aureus*. The best efficacy against both bacterial strains resulted from the polyglycidol with statistic distribution of the cationic and hydrophobic residues with polymer **6**, showing also the best selectivity to differentiate between mammalian cells and bacterial cell walls compared with the other polymers tested in this study. 

When cationic and hydrophobic residues are located in the same polyglycidol repeating unit being connected by a spacer, the impact on the cell envelope of *S. aureus* is significantly less effective than against *E. coli*. The outer part of the cell envelope of Gram-positive bacteria is composed of a thick peptidoglycan layer with integrated lipoteichoic acids followed by the inner cell membrane. Here, a sieving effect might be discussed, retarding the interaction of this polymer with the underlying cell membrane. Additionally, the vicinity of the cationic and hydrophobic groups in these polyglycidols compared to those prepared with statistic distribution of cationic and hydrophobic residues might disturb the interaction cascade of amphipathic polymers with the components of the cell envelope.

Changing the hydrophilic–lipophilic balance (HLB) to a higher lipophilic–cationic ratio in amphipathic polyglycidol with statistically distributed cationic and hydrophobic residues leads to a decrease in efficacy in the same order of magnitude against both bacterial strains *E. coli* and *S. aureus*. Antimicrobial efficacy of an amphipathic polymer strongly depends on its HLB, the microstructure induced by the type of polymer backbone, and the distribution and kind of the cationic and hydrophobic functionalities. 

## Figures and Tables

**Table 1:** Minimal inhibitory concentration against *E. coli* and *S. aureus* and hemolytic activity of functional polyglycidols with defined microstructures P(G^TMAPA^_15_-*co*-G^DDA^_12_) (**5**), P(G^APDEMA^_16_-*co*-G^DDA^_11_) (**6**), P(G^DDAc, q^)_27_ (**10**), P(G^TMAPA^_14_-*co*-G^DDADDAc^_13_) (**12**).

		*M*_n,NMR_^a^ (g·mol^−1^)	MIC_100_^b,c^ (µg/mL)		HC_50_^d^ (µg/mL)
Polymer	Microstructure		*E. coli*	*S. aureus*	
5	P(G^TMAPA^_15_-*co*-G^DDA^_12_)	10,111	30	20	>>500 ^f^
6	P(G^APDEMA^_16_-*co*-G^DDA^_11_)	9608	30	30	100
10	P(G^DDAc, q^)_27_	18,947	30	>200	<10
12	P(G^TMAPA^_14_-*co*-G^DDADDAc^_13_)	13,476	200 ^d^	>200‒500 ^e^	<10

^a^ Molecular weight (M_n,NMR_) calculated from ^1^H NMR with an accuracy of integration of ±5%; ^b^ Minimum inhibitory concentration which prevents the visible growth of 100% of the bacteria within 20 h monitoring time; ^c^ Inoculum size = 1 × 10^6^–2 × 10^6^ CFU·mL^−1^; ^d^ HC_50_: concentration causing 50% lysis (hemolysis) of red blood cells (RBCs) relative to Triton X-100 (positive control, 100%), linear polyglycidol PG_27_ (**1**) HC_50_ >> 500 µg/mL; ^e^ Non-growth value due to turbidity of solution caused by the polymer; ^f^ 10–500 µg/mL of polymer **5** causing agglutination of RBC, and about 6% (at 10 µg/mL)–40% (at 500 µg/mL) hemolysis.

## Supplementary Materials

The following are available online at www.mdpi.com/2073-4360/10/1/96/s1, Figure S1: ^1^H NMR spectrum of PG_27_ (**1**) measured in DMSO-*d*_6_, Figure S2: ^13^C NMR spectrum of PG_27_ (**1**) measured in DMSO-*d*_6_, Figure S3: DMF-SEC traces of PG_27_ (**1**), Scheme S1: Synthetic pathway to P(G^TMAPA^_15_-*co*-G^DDA^_12_) (**5***)***,** P(G^APDEMA^_16_-*co*-G^DDA^_11_) (**6**), P(G^DDAc, q^)_27_ (**10**) and P(G^TMAPA^_14_-*co*-G^DDADDAc^_13_) (**12**), Figure S4: ^1^H NMR spectrum of P(G^PC^)_27_ (**2**) measured in CDCl_3_, Figure S5: ^13^C NMR spectrum of P(G^PC^)_27_ (**2**) measured in CDCl_3_, Figure S6: DMF-SEC traces of P(G^PC^)_27_ (**2**), Figure S7: ^1^H NMR spectrum of P(G^DMAPA^_15_-*co*-G^DDA^_12_) (**3**) measured in CDCl_3_, Figure S8: ^13^C NMR spectrum of P(G^DMAPA^_15_-*co*-G^DDA^_12_) (**3**) measured in CDCl_3_, Figure S9: DMF-SEC traces of P(G^DMAPA^_15_-*co*-G^DDA^_12_) (**3**), Figure S10: ^1^H NMR spectrum of P(G^APDEA^_16_-*co*-G^DDA^_11_) (**4**) measured in MeOD, Figure S11: ^13^C NMR spectrum of P(G^APDEA^_16_-*co*-G^DDA^_11_) (**4**) measured in MeOD, Figure S12: DMF-SEC traces of P(G^APDEA^_16_-*co*-G^DDA^_11_) (**4**), Figure S13: ^13^C NMR spectrum of P(G^TMAPA^_56_-*co*-G^DDA^_12_) (**5**) measured in DMSO-*d*_6_, Figure S14: DMF-SEC traces of P(G^TMAPA^_56_-*co*-G^DDA^_12_) (**5**), Figure S15: ^13^C NMR spectrum of P(G^APDEMA^_16_-*co*-G^DDA^_11_) (**6**) measured in MeOD, Figure S16: ^1^H NMR spectrum of P(G^NPC^)_27_ (**7**) measured in DMSO-*d*_6_, Figure S17: ^13^C NMR spectrum of P(G^NPC^)_27_ (**7**) measured in DMSO-*d*_6_, Figure S18: DMF-SEC traces of P(G^NPC^)_27_ (**7**), Figure S19: ^1^H NMR spectrum of P(G^HCTL^)_27_ (**8**) measured in DMSO-*d*_6_, Figure S20: ^13^C NMR spectrum of P(G^HCTL^)_27_ (**8**) measured in DMSO-*d*_6_, Figure S21: DMF-SEC traces of P(G^HCTL^)_27_ (**8**), Figure S22: ^1^H NMR spectrum of P(G^DDAc^)_27_ (**9**) measured in CDCl_3_. Figure S23: ^13^C NMR spectrum of P(G^DDAc^)_27_ (**9**) measured in CDCl_3_, Figure S24: DMF-SEC traces of P(G^DDAc^)_27_ (**9**), Figure S25: ^13^C NMR spectrum of P(G^DDAc, q^)_27_ (**10**) measured in CDCl_3_, Figure S26: DMF-SEC traces of P(G^DDAc, q^)_27_ (**10**), Figure S27: ^1^H NMR spectrum of P(G^DMAPA^_14_-*co*-G^DDADDAc^_13_) (**11**) measured in CDCl_3_/acetone-*d*_6_, Figure S28: ^13^C NMR spectrum of P(G^DMAPA^_14_-*co*-G^DDADDAc^_13_) (**11**) measured in CDCl_3_/acetone-*d*_6_, Figure S29: DMF-SEC traces of P(G^DMAPA^_14_-*co*-G^DDADDAc^_13_) (**11**), Figure S30: ^13^C NMR spectrum of P(G^TMAPA^_14_-*co*-G^DDADDAc^_13_) (**12**) measured in CDCl_3_/acetone-*d*_6_, Figure S31: DMF-SEC traces of P(G^TMAPA^_14_-*co*-G^DDADDAc^_13_) (**12**).
