# Supplementary material for: Novel Antibacterial Polyglycidols: Relationship between Structure and Properties
Source: Polymers (Basel). 2018 Jan 20;10(1):96. doi: 10.3390/polym10010096 (PMC6414948; doi:10.3390/polym10010096)
Supplement: Supplementary file 1 [file polymers-10-00096-s001.pdf]

Supporting Information

Novel antimicrobial polyglycidols: relationship between structure and properties

Fabian Marquardt, Cornelia Stöcker, Rita Gartzen, Elisabeth Heine, Helmut Keul*, Martin Möller*

Institute of Technical and Macromolecular Chemistry, RWTH Aachen University and DWI-Leibniz-Institute for Interactive Materials, Forckenbeckstr. 50, D-52056 Aachen, Germany; Marquardt@dwi.rwth-aachen.de (F. M.); Cornelia.stoecker@rwth-aachen.de (C. S.); Gartzen@dwi.rwth-aachen.de (R. G.); Heine@dwi.rwth-aachen.de (E. H.)
* Correspondence: Keul@dwi.rwth-aachen.de (H. K.); Moeller@dwi.rwth-aachen.de (M. M.)

1. Synthesis of linear polyglycidol (1)

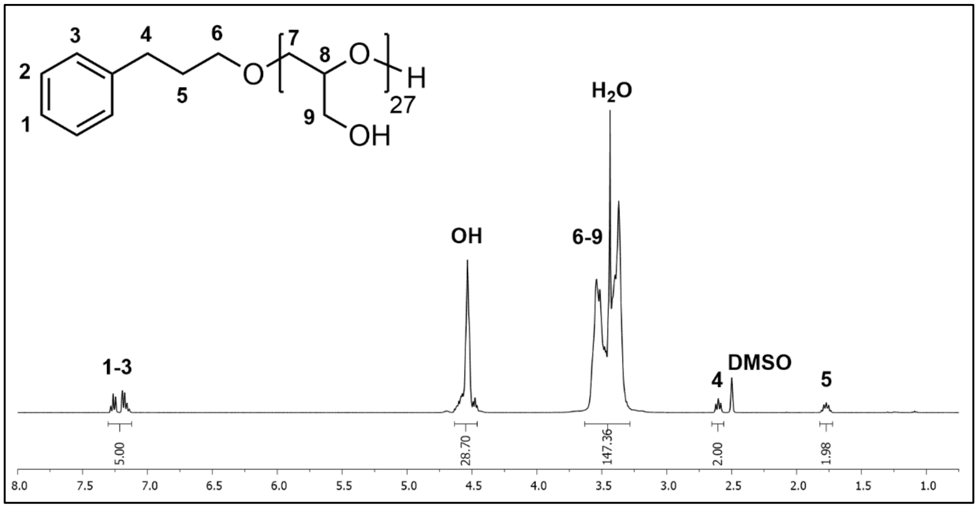

Figure S1. ^{1}H NMR spectrum of PG_{27} (**1**) measured in DMSO-d_6.

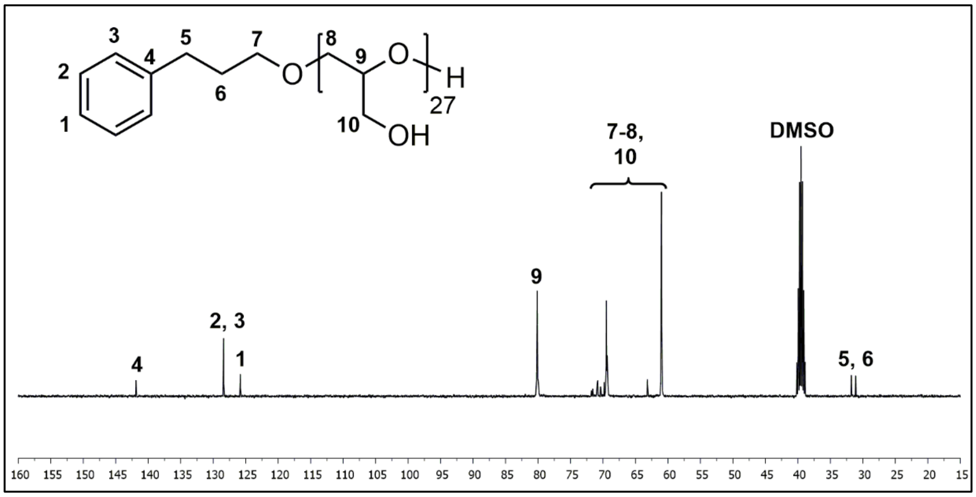

Figure S2. ^{13}C NMR spectrum of PG_{27} (**1**) measured in DMSO-d_6.

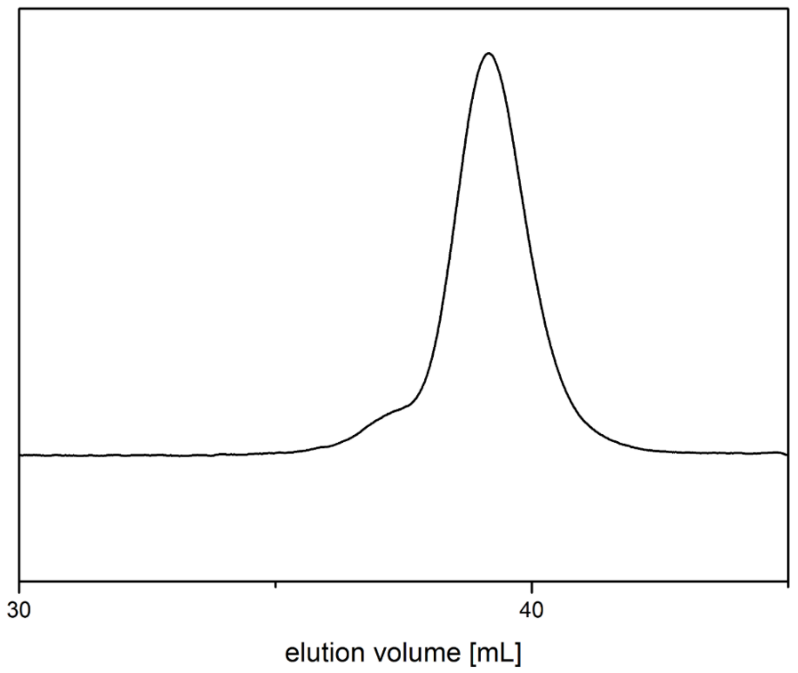

Figure S3. DMF-SEC traces of PG_{27} (**1**).

2. Synthetic pathway

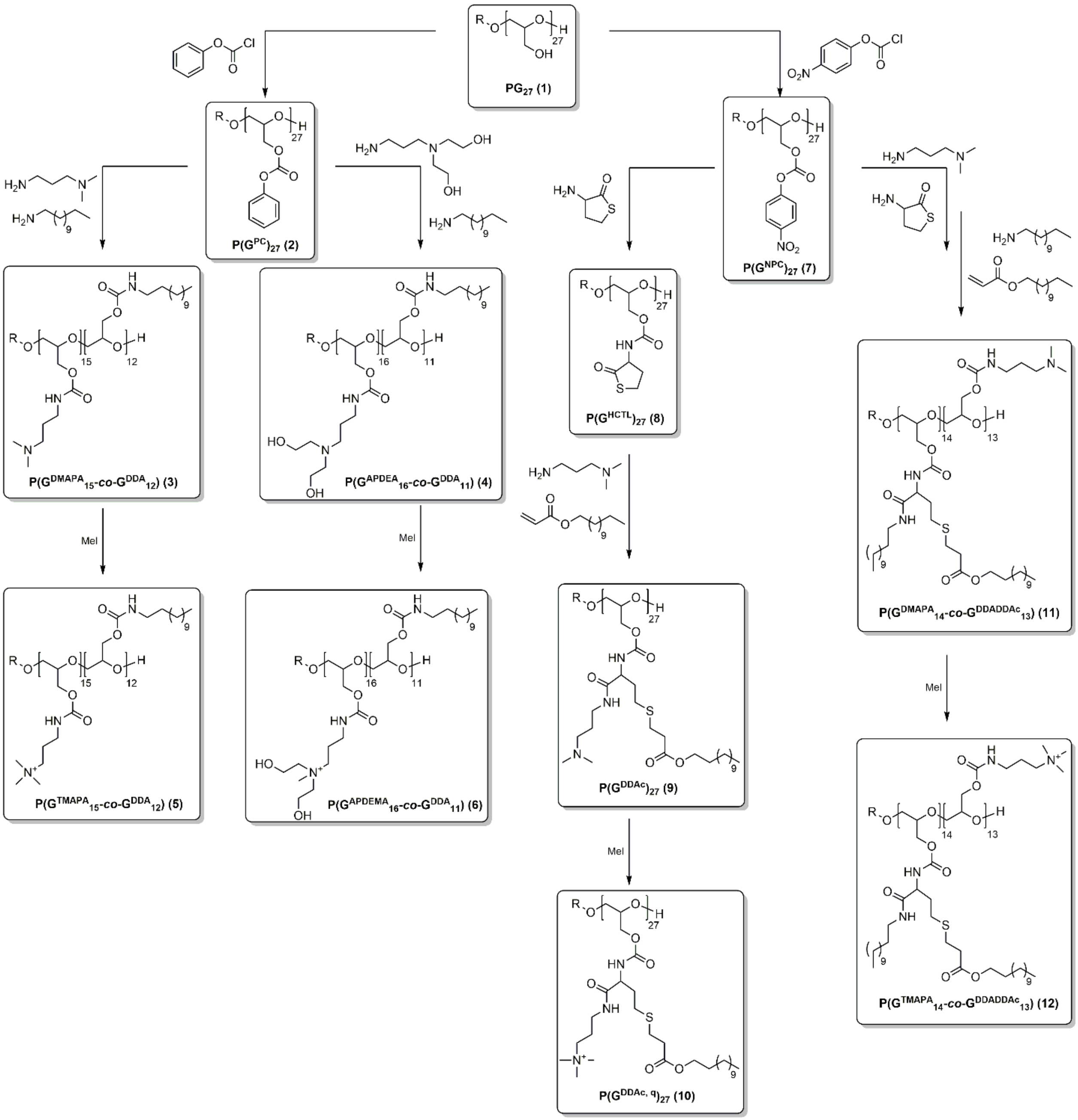

Scheme S1: Synthetic pathway to P(G^{TMAPA}_{15}-*co*-G^{DDA}_{12}) (**5**)**,** P(G^{APDEMA}_{16}-*co*-G^{DDA}_{11}) (**6**)**,** P($G^{DDAc,\ q}$)$_{27}$ (**10**) and P(G^{TMAPA}_{14}-*co*-$G^{DDADDAc}_{13}$) (**12**).

3. Synthesis of P(G^{TMAPA}$_{15}$-*co*-G^{DDA}$_{12}$) (5) and P(G^{APDEMA}$_{16}$-*co*-G^{DDA}$_{11}$) (6)

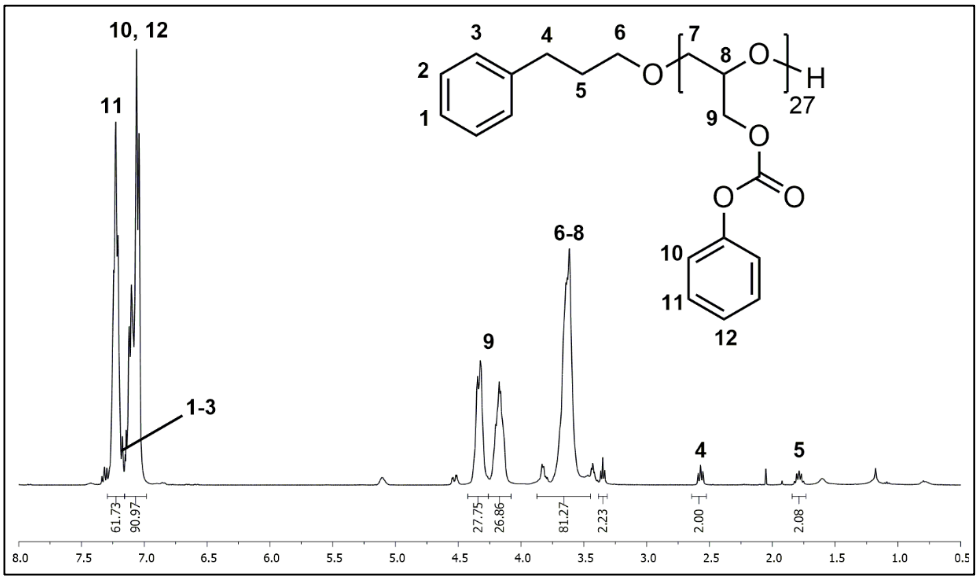

Figure S4. ^{1}H NMR spectrum of P(G^{PC})$_{27}$ (**2**) measured in $CDCl_3$.

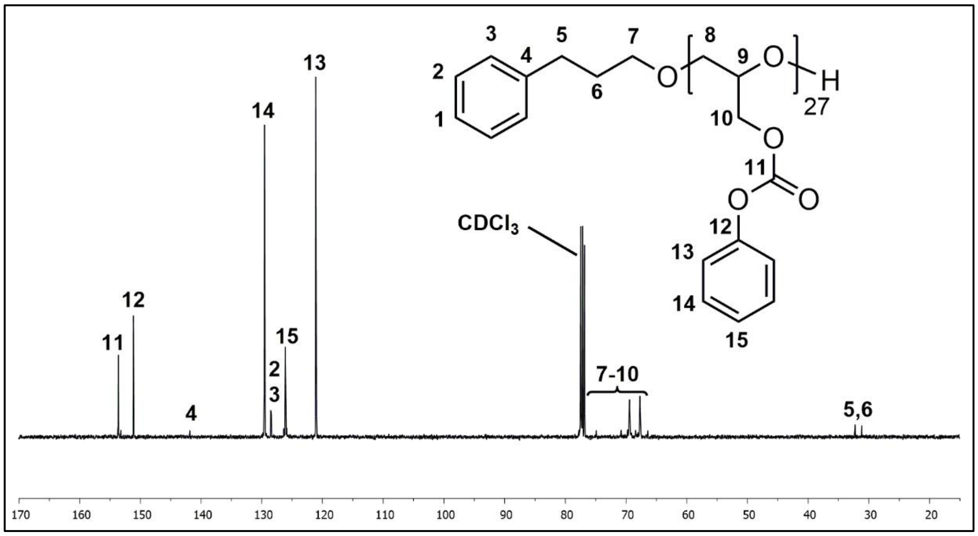

Figure S5. ^{13}C NMR spectrum of P(G^{PC})$_{27}$ (**2**) measured in $CDCl_3$.

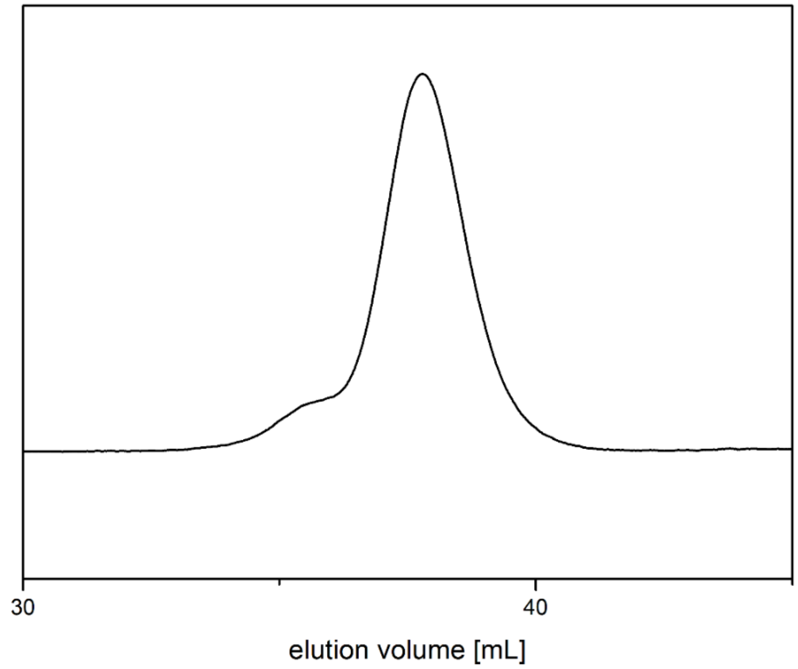

Figure S6. DMF-SEC traces of P(G^{PC})$_{27}$ (**2**).

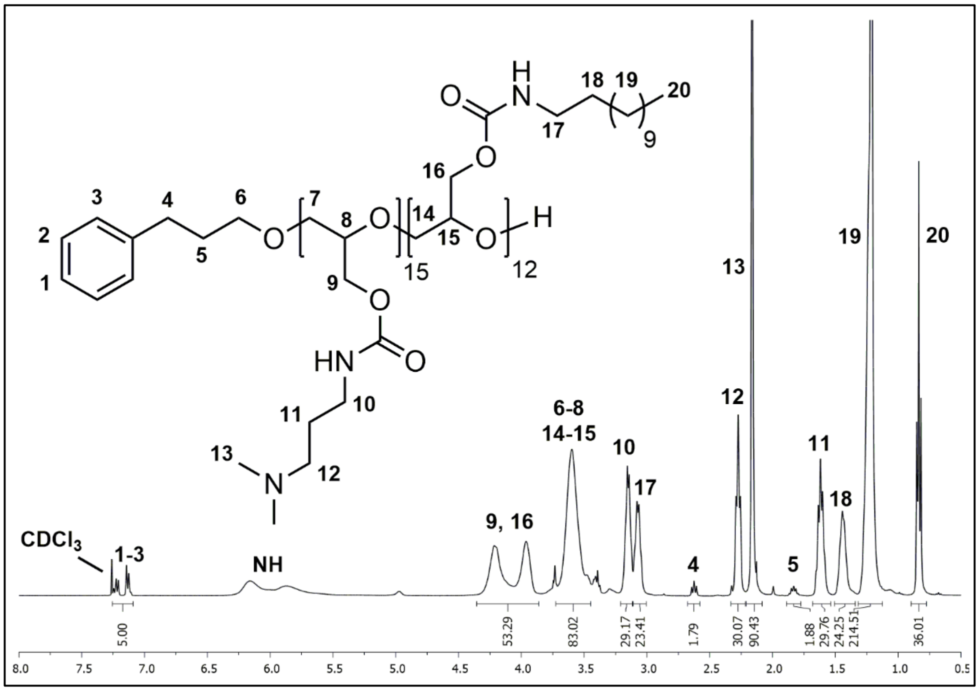

Figure S7. ^{1}H NMR spectrum of P($G^{DMAPA}{}_{15}$-*co*-$G^{DDA}{}_{12}$) (**3**) measured in $CDCl_3$.

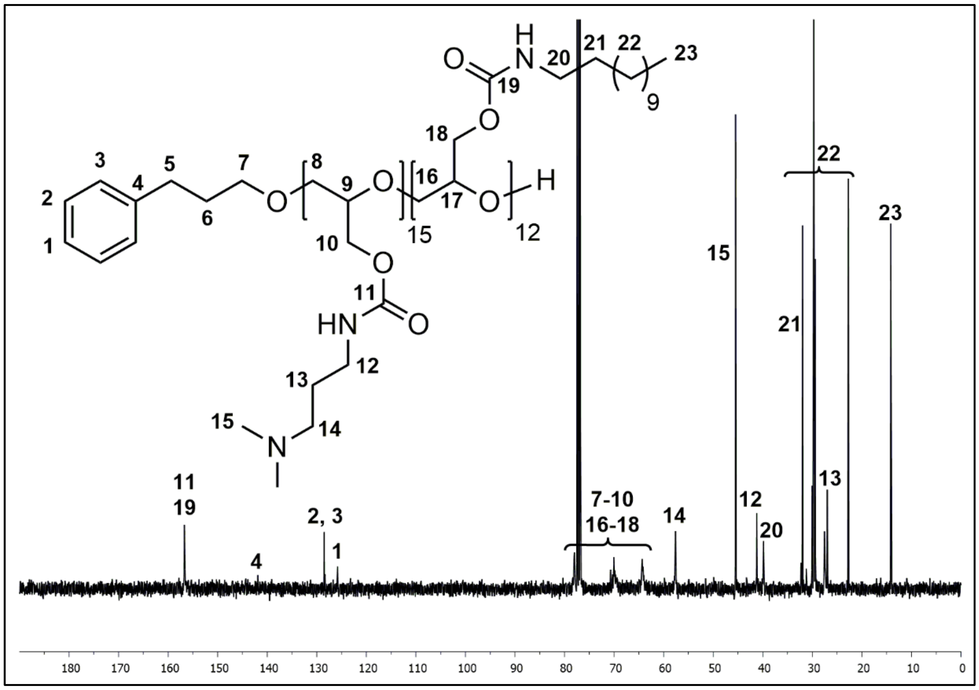

Figure S8. ^{13}C NMR spectrum of P(G^{DMAPA}$_{15}$-*co*-G^{DDA}$_{12}$) (**3**) measured in $CDCl_3$.

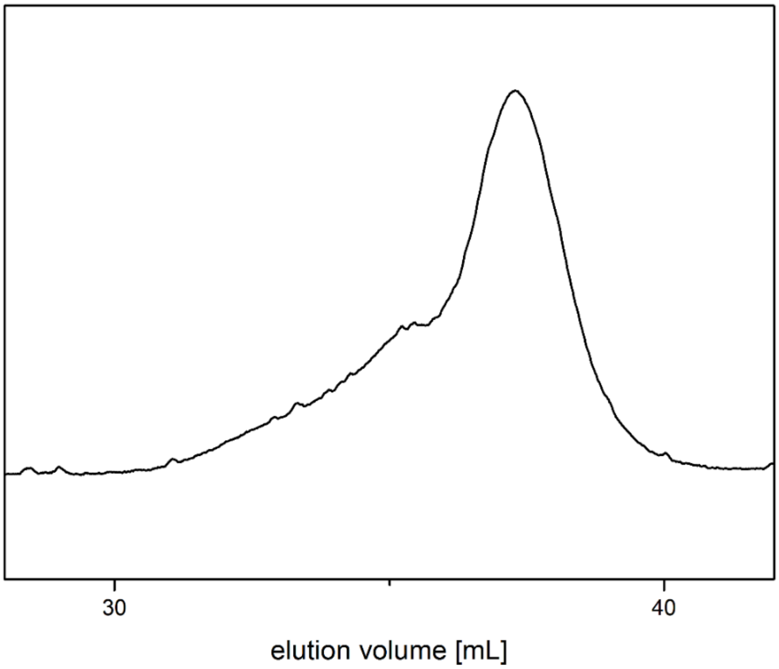

Figure S9. DMF-SEC traces of P(G^{DMAPA}$_{15}$-*co*-G^{DDA}$_{12}$) (**3**).

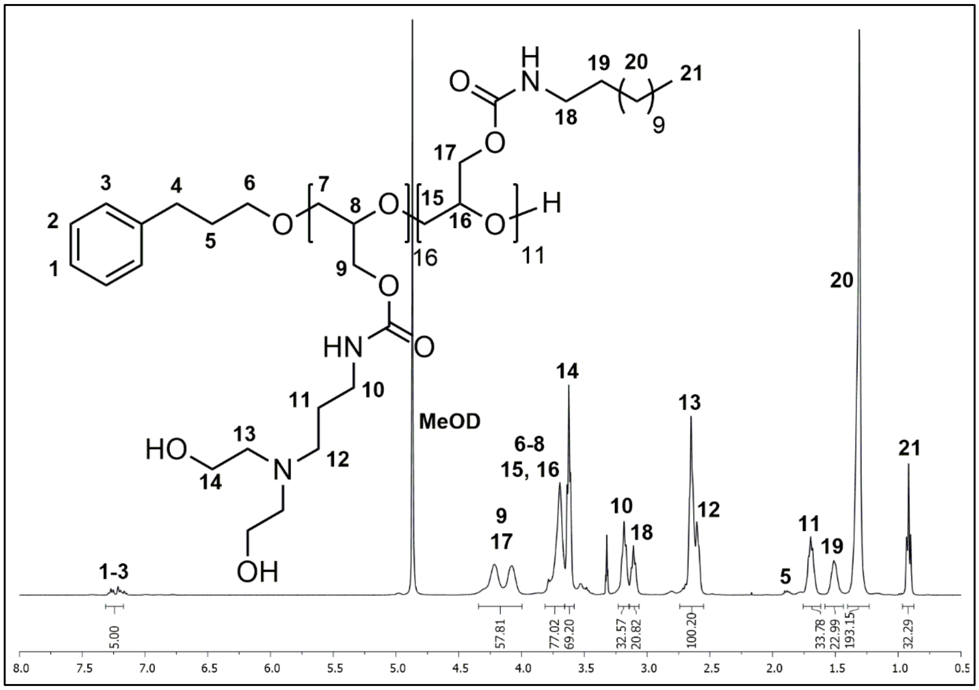

Figure S10. ^{1}H NMR spectrum of P(G^{APDEA}$_{16}$-*co*-G^{DDA}$_{11}$) (**4**) measured in MeOD.

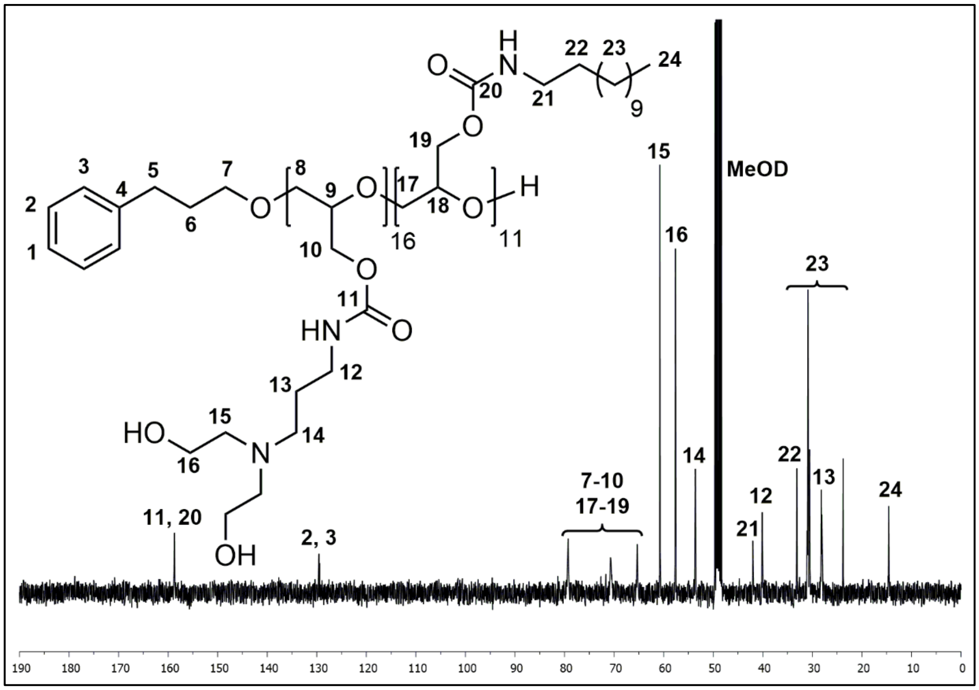

Figure S11. ^{13}C NMR spectrum of P(G^{APDEA}$_{16}$-*co*-G^{DDA}$_{11}$) (**4**) measured in MeOD.

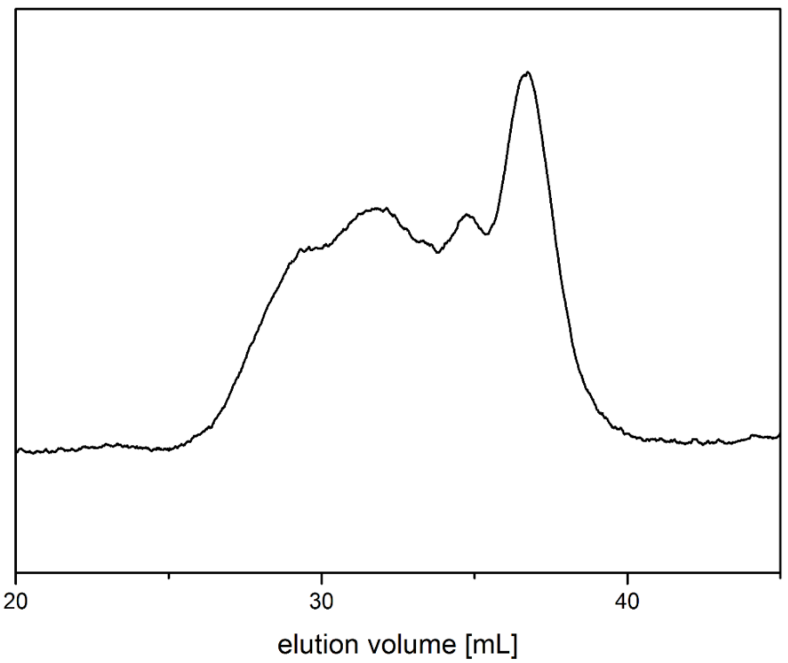

Figure S12. DMF-SEC traces of P(G^{APDEA}$_{16}$-*co*-G^{DDA}$_{11}$) (**4**).

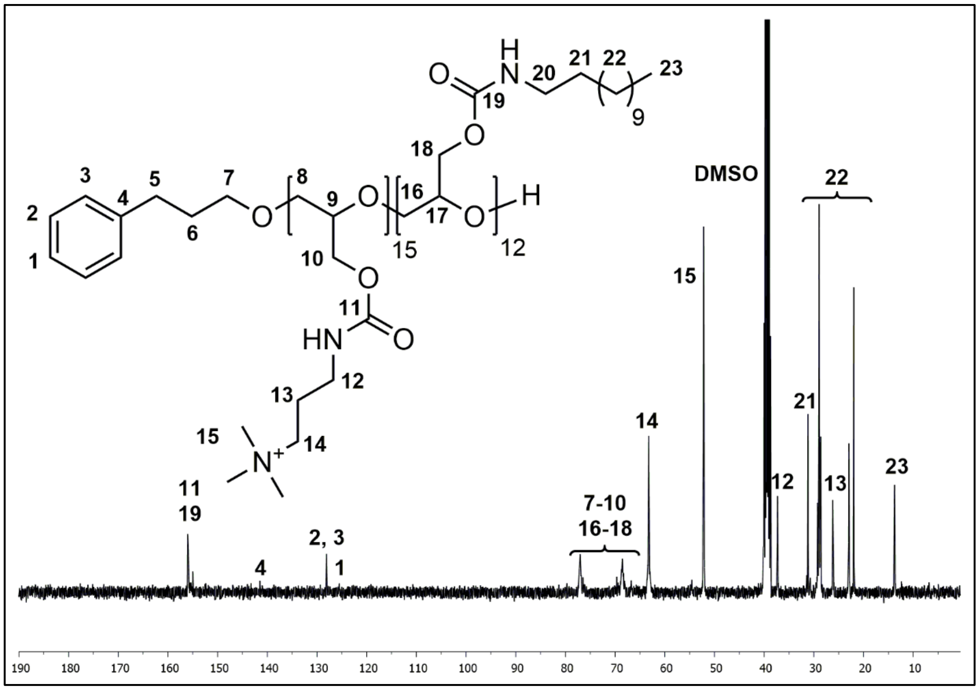

Figure S13. ^{13}C NMR spectrum of P(G^{TMAPA}$_{15}$-*co*-G^{DDA}$_{12}$) (**5**) measured in DMSO-*d*$_6$.

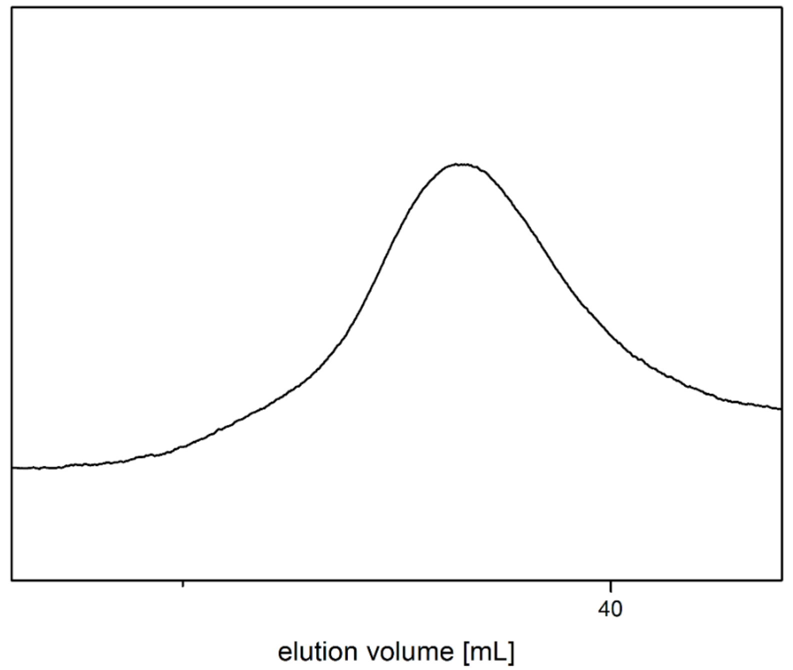

Figure S14. DMF-SEC traces of P($G^{TMAPA}{}_{15}$-*co*-$G^{DDA}{}_{12}$) (**5**).

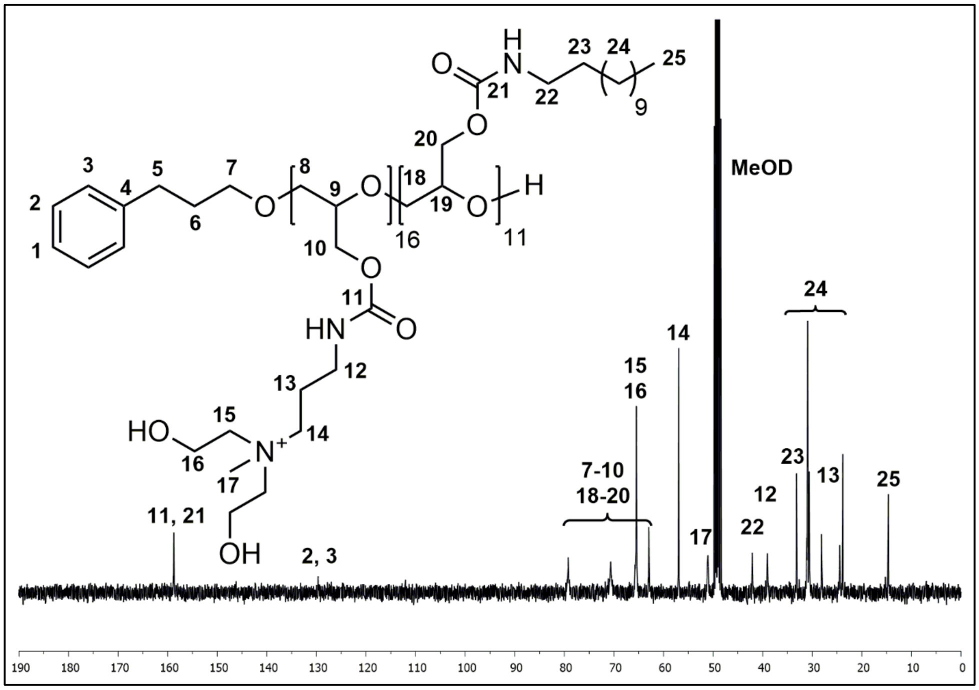

Figure S15. ^{13}C NMR spectrum of P($G^{APDEMA}{}_{16}$-*co*-$G^{DDA}{}_{11}$) (**6**) measured in MeOD.

4. Synthesis of P(G$^{DDAc, q}$)$_{27}$ (10)

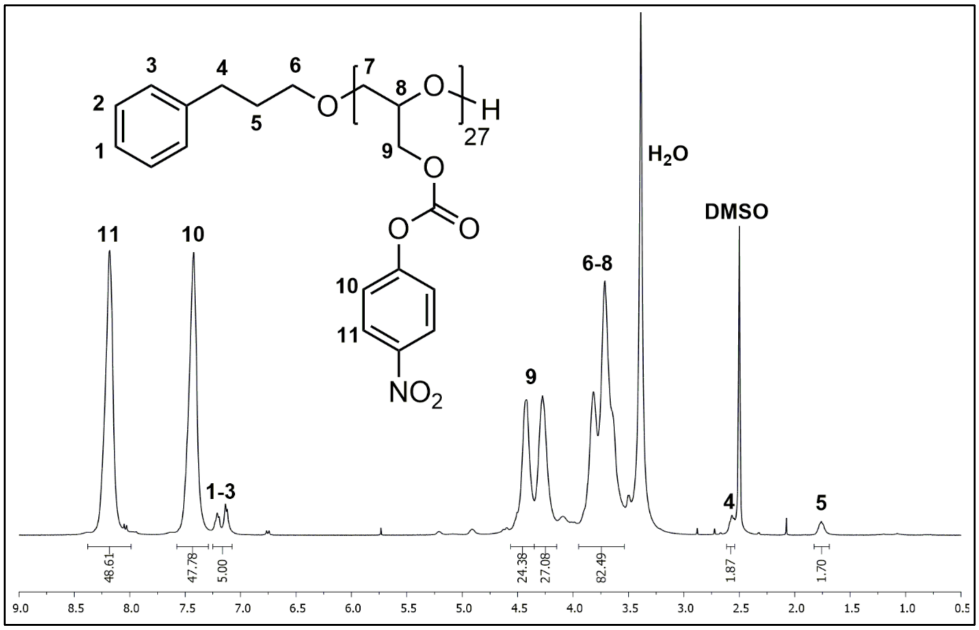

Figure S16. ^{1}H NMR spectrum of P(G^{NPC})$_{27}$ (**7**) measured in DMSO-*d*$_6$.

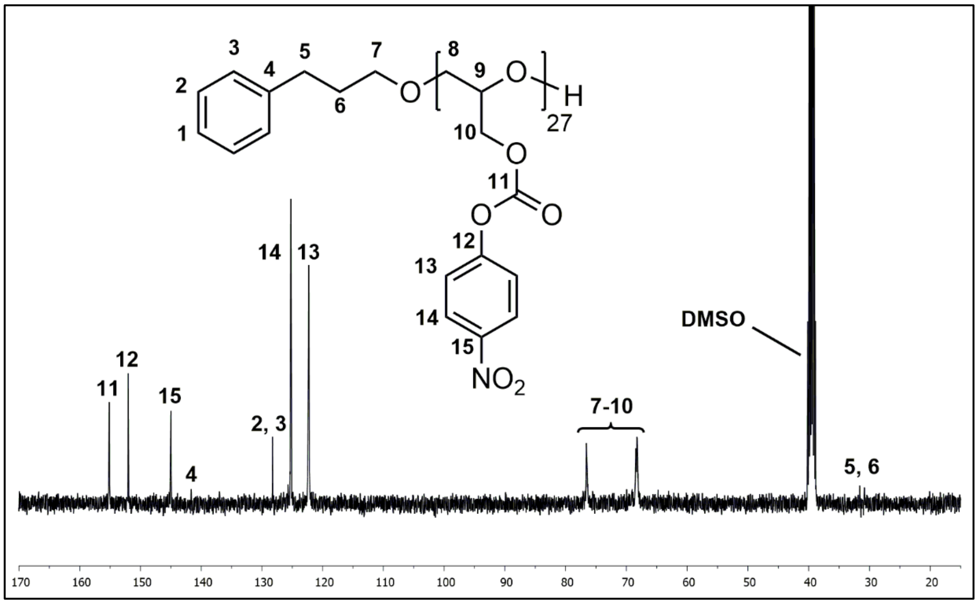

Figure S17. ^{13}C NMR spectrum of P(G^{NPC})$_{27}$ (**7**) measured in DMSO-*d*$_6$.

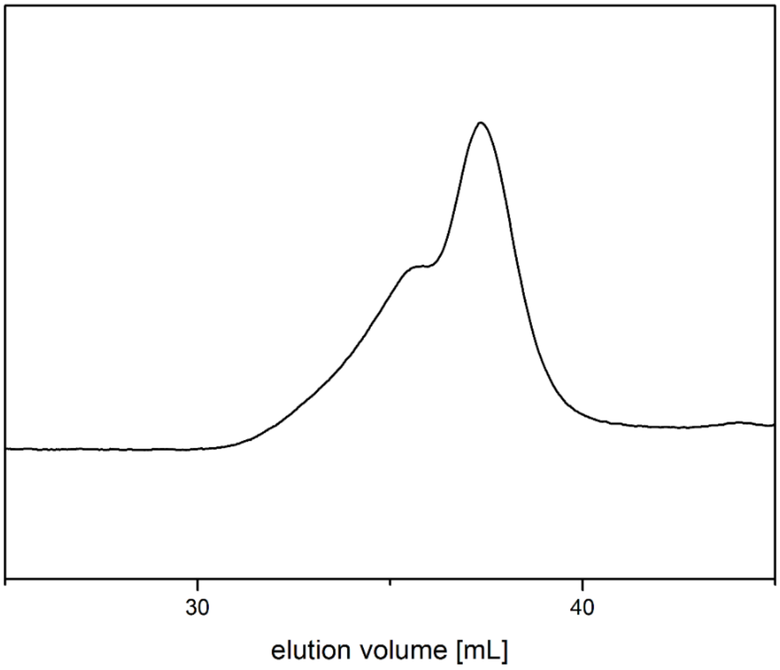

Figure S18. DMF-SEC traces of $P(G^{NPC})_{27}$ (**7**).

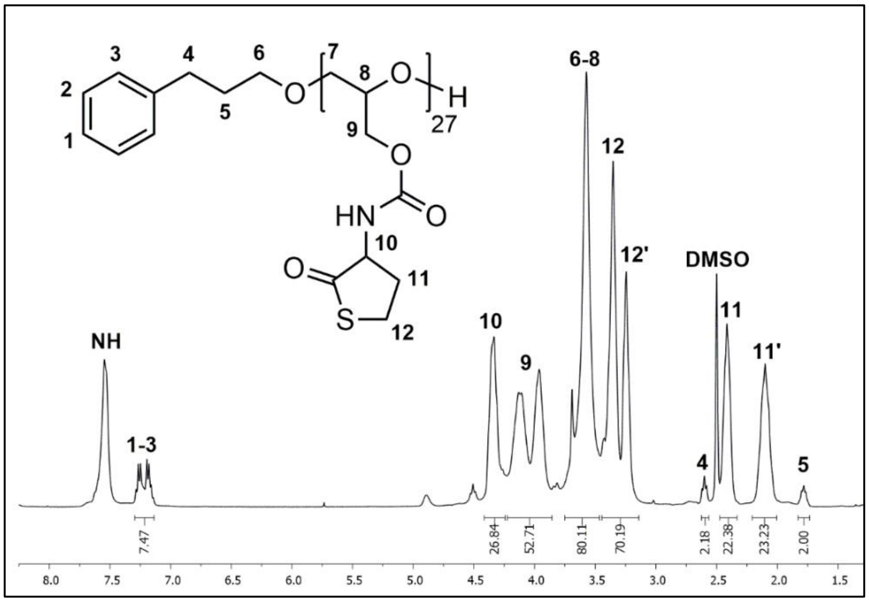

Figure S19. ^{1}H NMR spectrum of $P(G^{HCTL})_{27}$ (**8**) measured in DMSO-d_6.

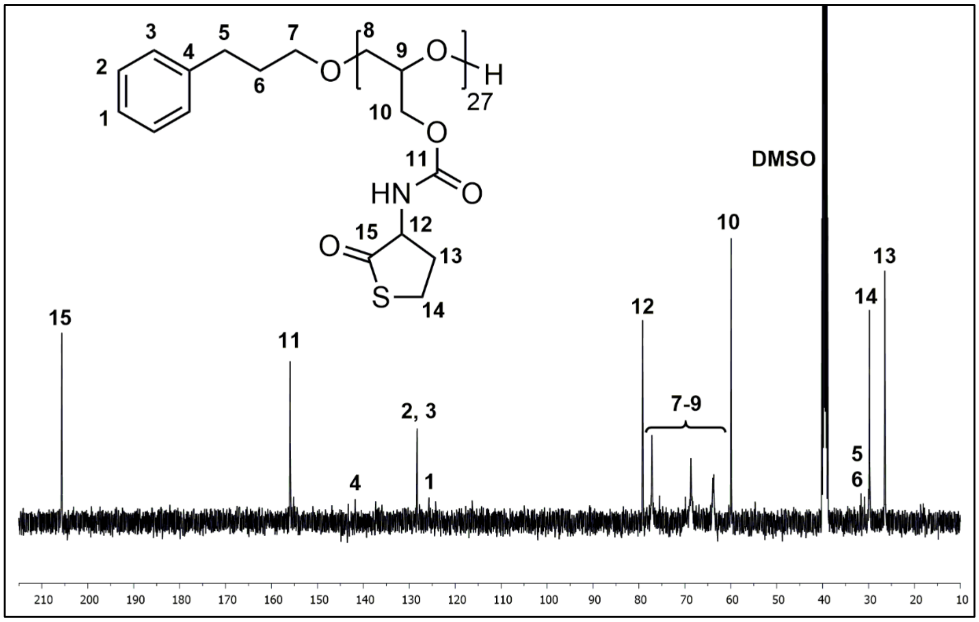

Figure S20. ^{13}C NMR spectrum of P(G^{HCTL})$_{27}$ (**8**) measured in DMSO-d_6.

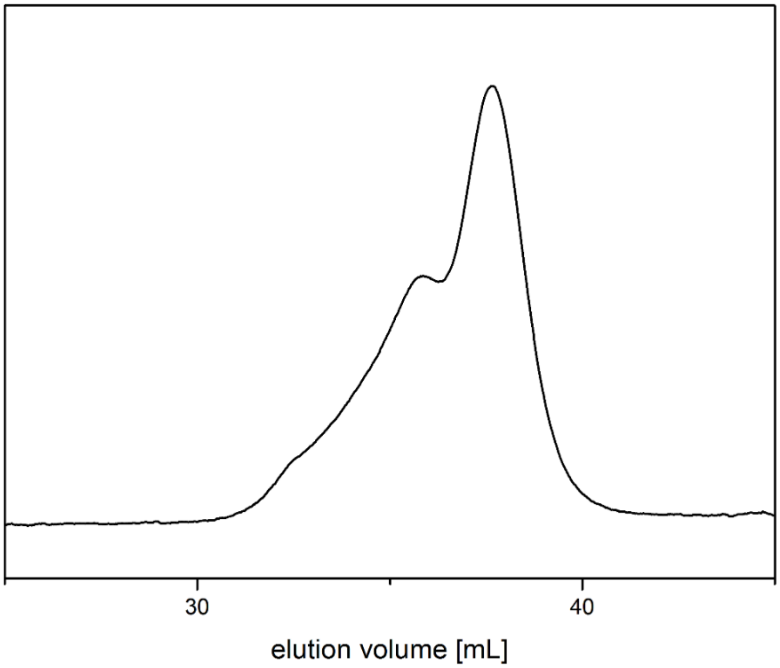

Figure S21. DMF-SEC traces of P(G^{HCTL})$_{27}$ (**8**).

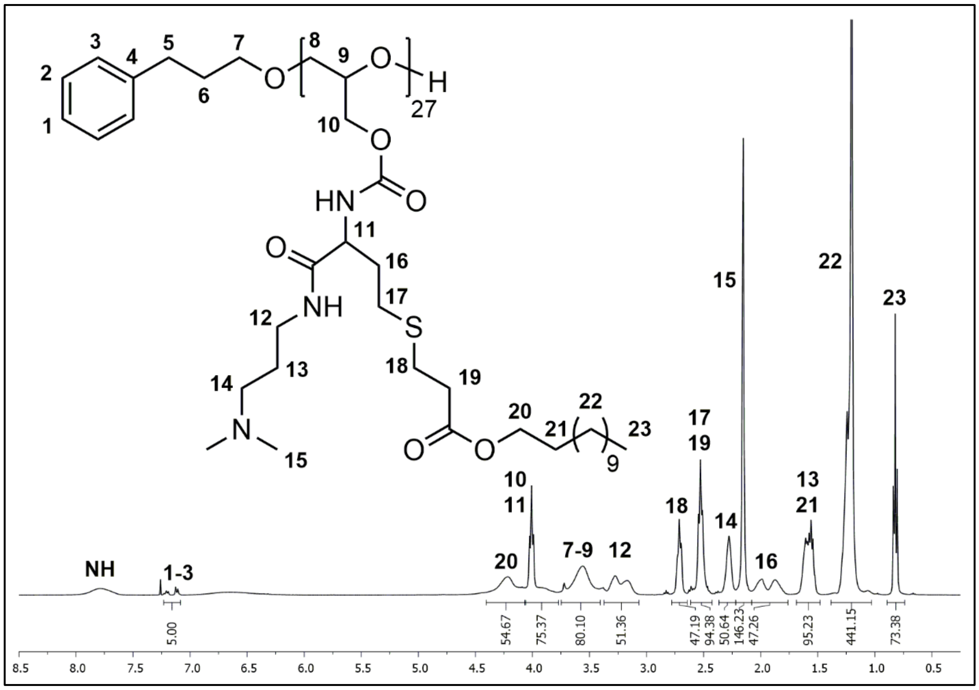

Figure S22. ^{1}H NMR spectrum of P(G^{DDAc})$_{27}$ (**9**) measured in $CDCl_3$.

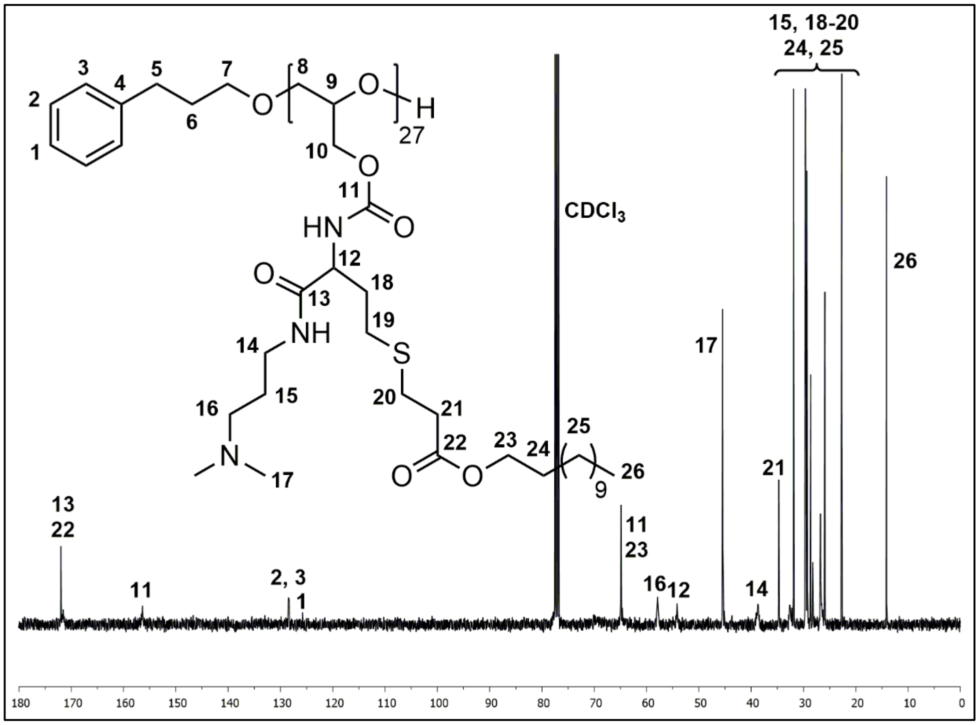

Figure S23. ^{13}C NMR spectrum of P(G^{DDAc})$_{27}$ (**9**) measured in $CDCl_3$.

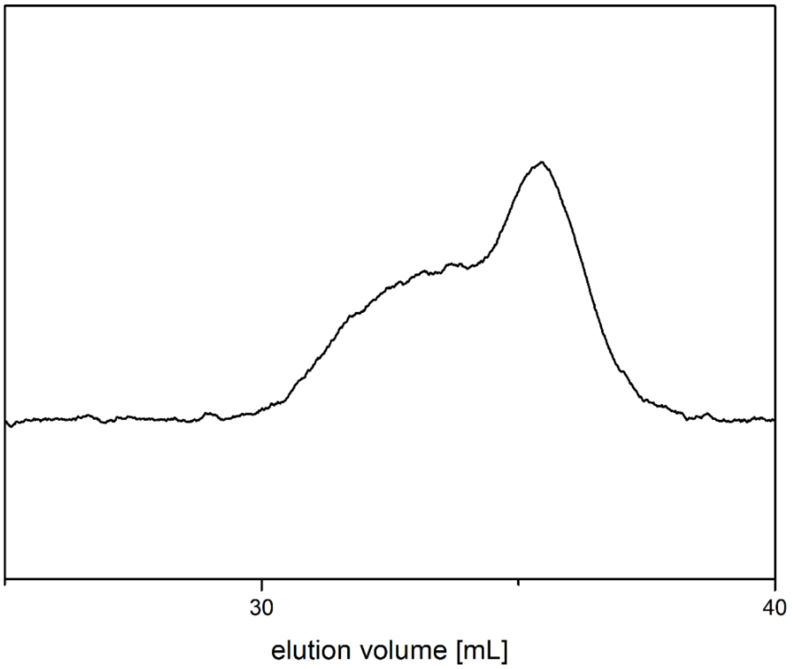

Figure S24. DMF-SEC traces of P(G^{DDAc})$_{27}$ (**9**).

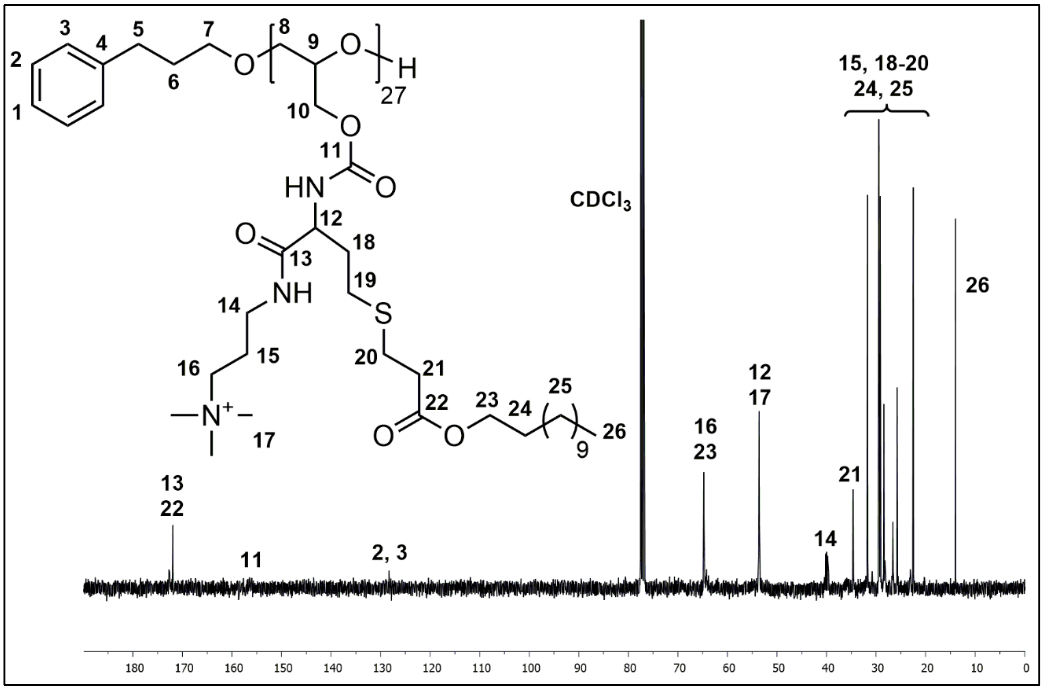

Figure S25. ^{13}C NMR spectrum of P(G$^{DDAc, q}$)$_{27}$ (**10**) measured in $CDCl_3$.

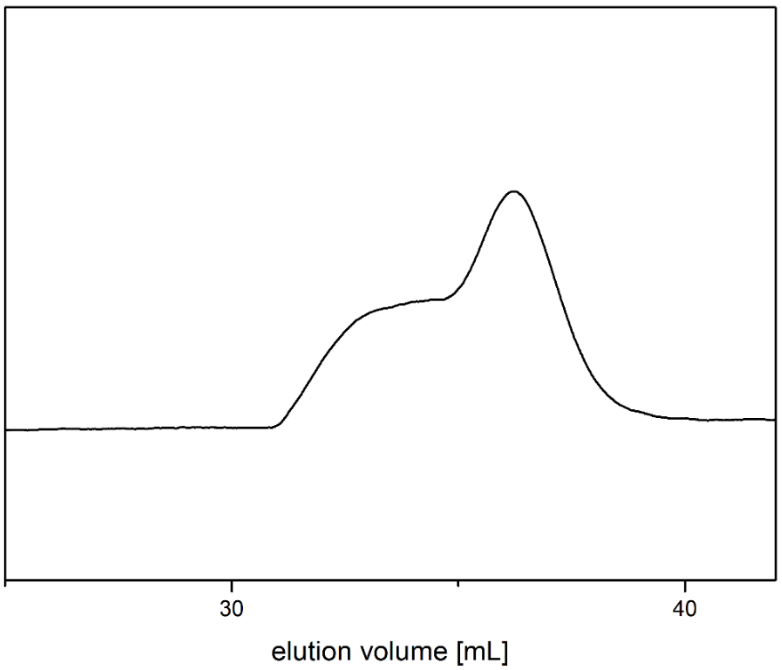

Figure S26. DMF-SEC traces of P($G^{DDAc,\ q}$)$_{27}$ (**10**).

5. Synthesis of P($G^{TMAPA}{}_{14}$-*co*-$G^{DDADDAc}{}_{13}$) (12)

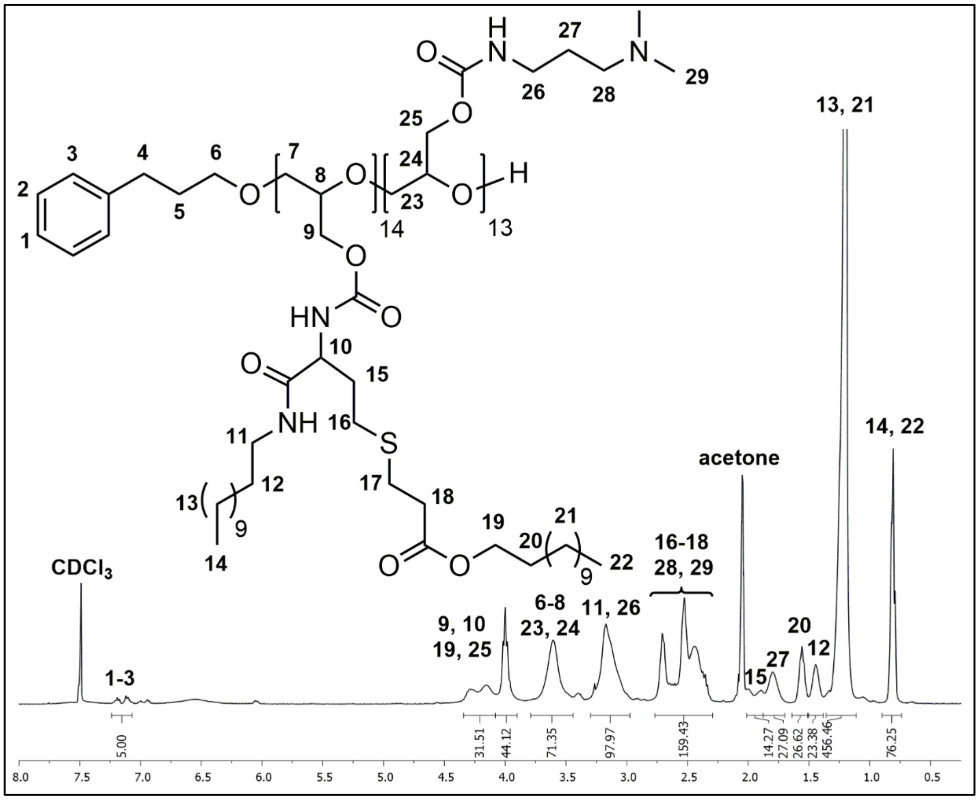

Figure S27. ^{1}H NMR spectrum of P($G^{DMAPA}{}_{14}$-*co*-$G^{DDADDAc}{}_{13}$) (**11**) measured in $CDCl_3$/acetone-d_6.

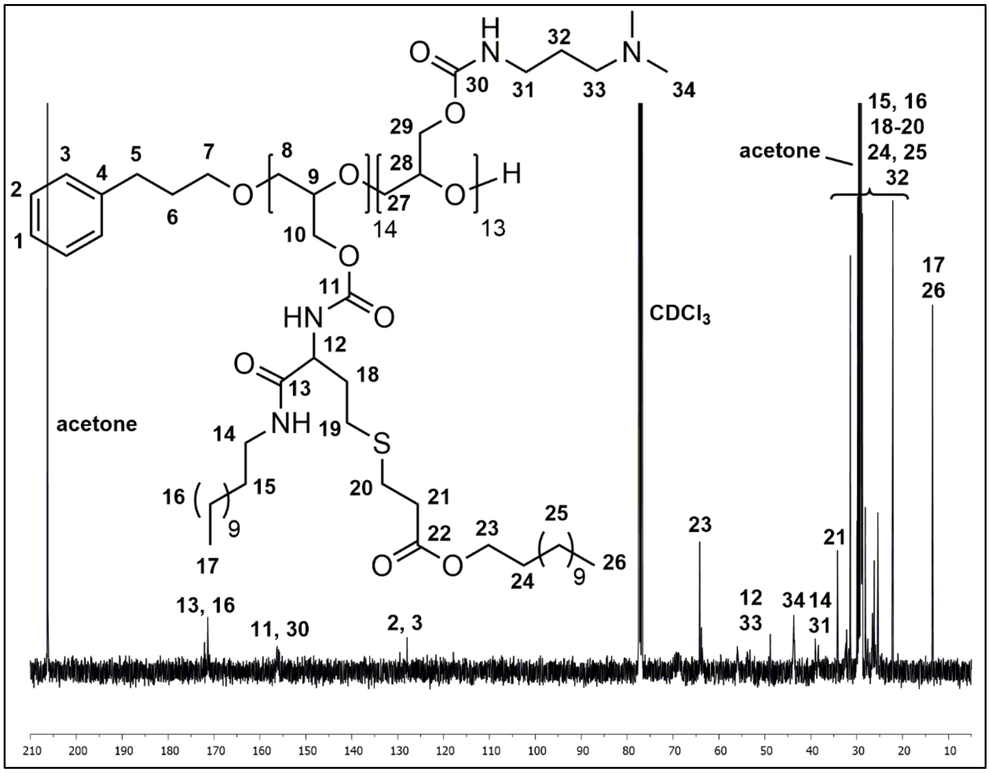

Figure S28. ^{13}C NMR spectrum of P(G^{DMAPA}$_{14}$-*co*-G^{DDADDAc}$_{13}$) (**11**) measured in CDCl$_3$/acetone-*d*$_6$.

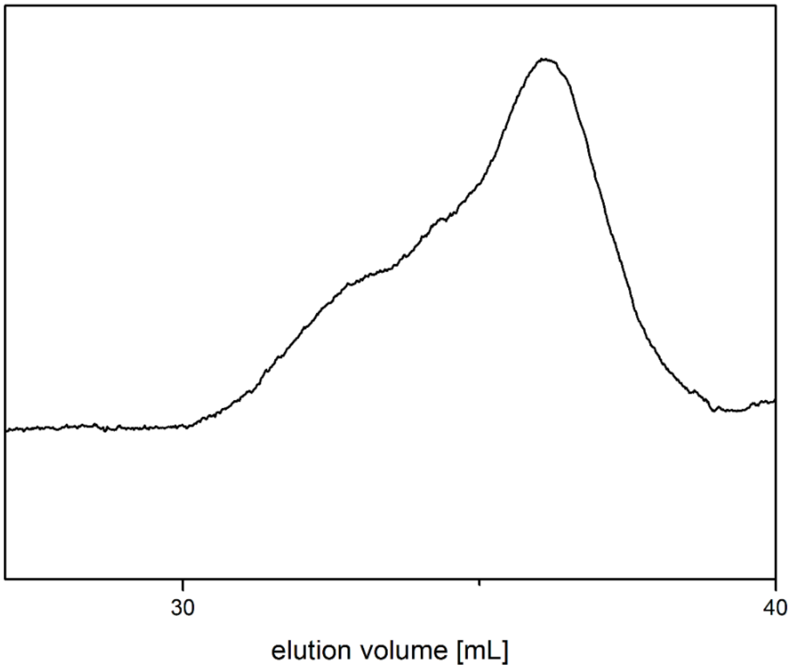

Figure S29. DMF-SEC traces of P(G^{DMAPA}$_{14}$-*co*-G^{DDADDAc}$_{13}$) (**11**).

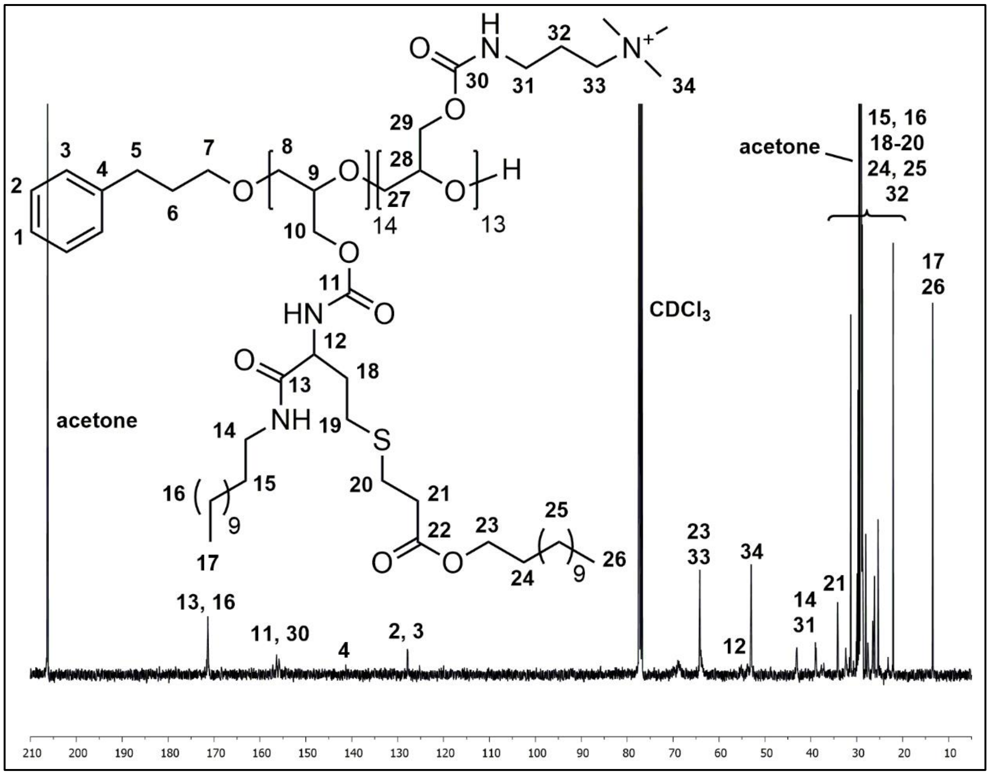

Figure S30. ^{13}C NMR spectrum of P(G$^{TMAPA}{}_{14}$-*co*-G$^{DDADDAc}{}_{13}$) (**12**) measured in CDCl$_3$/acetone-d_6.

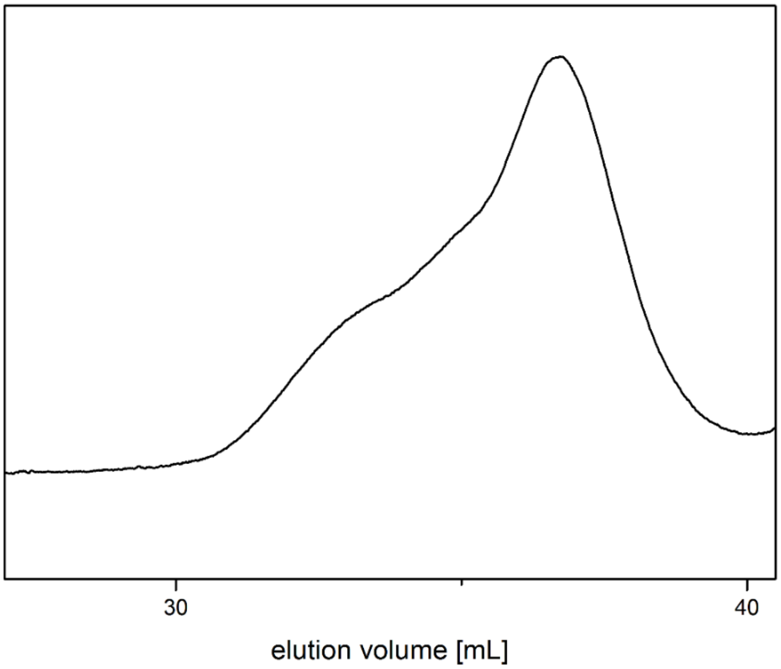

Figure S31. DMF-SEC traces of P(G$^{TMAPA}{}_{14}$-*co*-G$^{DDADDAc}{}_{13}$) (**12**).
